# Albumin uptake and processing by the proximal tubule: physiological, pathological, and therapeutic implications

**DOI:** 10.1152/physrev.00014.2021

**Published:** 2022-04-04

**Authors:** Bruce A. Molitoris, Ruben M. Sandoval, Shiv Pratap S. Yadav, Mark C. Wagner

**Affiliations:** ^1^Division of Nephrology, Department of Medicine, Indiana University School of Medicine, Indianapolis, Indiana; ^2^Department of Cellular and Integrative Physiology, Indiana University School of Medicine, Indianapolis, Indiana

**Keywords:** cubilin, drug delivery, endocytosis, FcRn, megalin, transcytosis

## Abstract

For nearly 50 years the proximal tubule (PT) has been known to reabsorb, process, and either catabolize or transcytose albumin from the glomerular filtrate. Innovative techniques and approaches have provided insights into these processes. Several genetic diseases, nonselective PT cell defects, chronic kidney disease (CKD), and acute PT injury lead to significant albuminuria, reaching nephrotic range. Albumin is also known to stimulate PT injury cascades. Thus, the mechanisms of albumin reabsorption, catabolism, and transcytosis are being reexamined with the use of techniques that allow for novel molecular and cellular discoveries. Megalin, a scavenger receptor, cubilin, amnionless, and Dab2 form a nonselective multireceptor complex that mediates albumin binding and uptake and directs proteins for lysosomal degradation after endocytosis. Albumin transcytosis is mediated by a pH-dependent binding affinity to the neonatal Fc receptor (FcRn) in the endosomal compartments. This reclamation pathway rescues albumin from urinary losses and cellular catabolism, extending its serum half-life. Albumin that has been altered by oxidation, glycation, or carbamylation or because of other bound ligands that do not bind to FcRn traffics to the lysosome. This molecular sorting mechanism reclaims physiological albumin and eliminates potentially toxic albumin. The clinical importance of PT albumin metabolism has also increased as albumin is now being used to bind therapeutic agents to extend their half-life and minimize filtration and kidney injury. The purpose of this review is to update and integrate evolving information regarding the reabsorption and processing of albumin by proximal tubule cells including discussion of genetic disorders and therapeutic considerations.

## Graphical abstract

**Figure:**
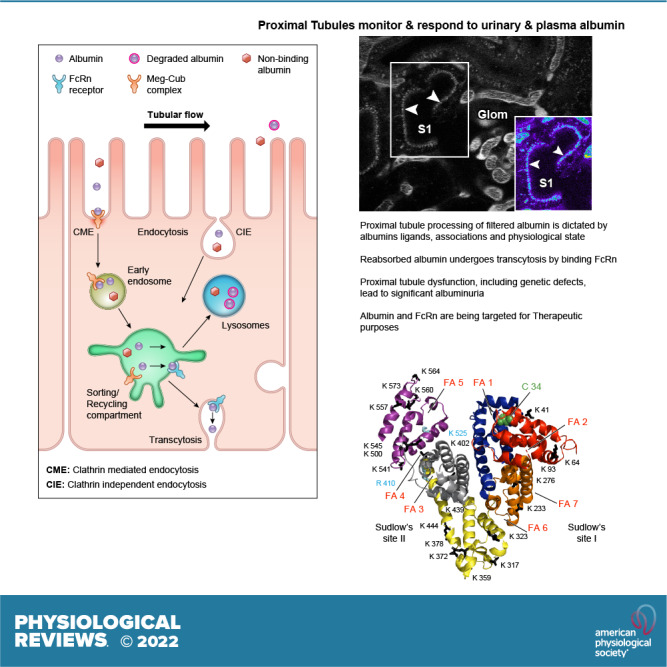

CLINICAL HIGHLIGHTS

1. The clinical relevance of proteinuria, and especially albuminuria, has been well documented in kidney and cardiovascular disease occurrence and progression.
2. The quantitative and mechanistic aspects and role of different contributing nephron components to albuminuria remains an area of considerable excitement. In particular, the role of proximal tubules in albumin reabsorption and reclamation, largely ignored for many years, is now known to be an important determinant of the urinary barrier to albuminuria under physiological and pathological conditions.
3. Understanding the role of the proximal tubule in albumin metabolism requires further investigation into the in vivo mechanisms of cellular uptake, intracellular trafficking, and transcytosis.
4. Whether and how albumin metabolism is impacted by albumin’s modifications and/or ligand association are also critical areas of investigation to determine whether these altered albumins are metabolized differently and are responsible for cell injury. This is especially important given data showing that modified albumins have altered vascular clearance and altered receptor binding.
5. The design and effectiveness of novel therapeutics to prevent and treat proximal tubule cell injury and chronic kidney disease will be more effective if proximal tubule albumin metabolism is fully understood.

## 1. INTRODUCTION

**FIGURE 1.**
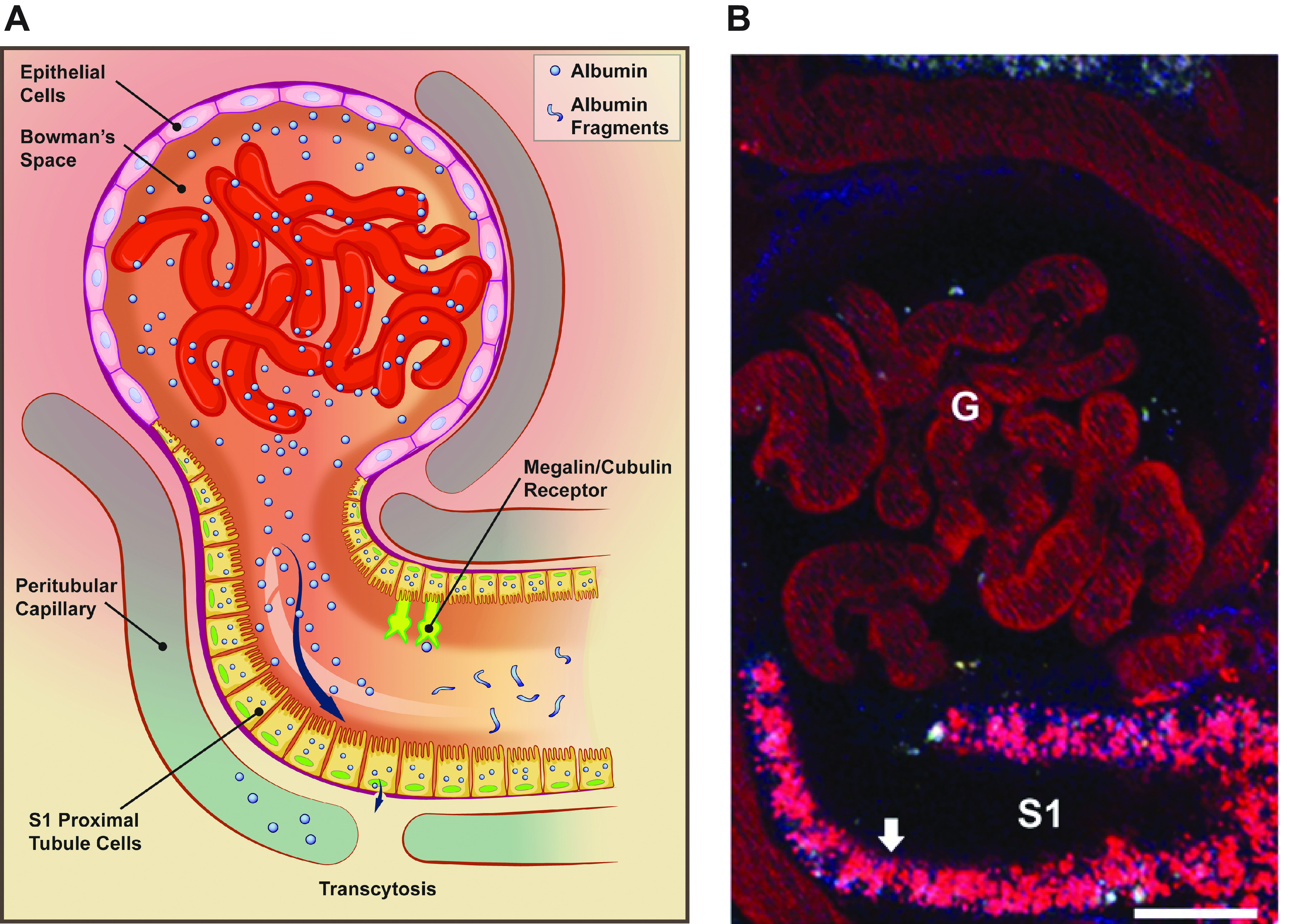
*A*: albumin uptake and processing by the proximal tubule. Albumin filtered across the glomerulus into Bowman’s space is reabsorbed after binding by the apical megalin-cubilin receptor complex. Both receptor-mediated endocytosis, via clathrin-coated vesicles, and fluid-phase endocytosis result in albumin reabsorption. After uptake, albumin can be transcytosed or undergo catabolism via lysosomal degradation. Albumin fragments in the urine result from lysosomal exocytosis of partially degraded albumin or peptide hydrolysis by apical membrane proteases. *B*: 25-μm 3-dimensional volume demonstrating Texas Red-labeled albumin endocytosed into proximal tubule cells, especially the S1 segment (S1). G, glomerular capillaries. Arrow indictes proximal tubule cells; bar = 20 μm. Figure modified from Ref. 2, with permission from the *Journal of the American Society of Nephrology*.

The role of the proximal tubule (PT) in albumin metabolism is an understudied and underappreciated area, especially in the clinical arena. Urinary albumin is thought by most to represent glomerular filtration of albumin, with little or no consideration given to the role of the PT in reabsorbing, catabolizing, and reclaiming albumin via transcytosis (1). As urinary albumin is a clinical risk factor for kidney disease development and progression and cardiovascular disease, the mechanism(s) mediating the presence and toxic effects of albuminuria, especially with respect to the PT, remain important questions. The quantitative role of the glomerular filtration barrier and the PT in albuminuria has been reevaluated. Glomerular permeability and proximal tubule reabsorption have essential roles in the renal handling and determination of albuminuria (2, 3). Evidence from multiple investigative teams suggests that filtration of albumin, under physiological conditions, is greater than previously determined (2) and the PT has an increased role in minimizing albuminuria through the reabsorption and transcytosis of albumin under both physiological and disease conditions. Proximal tubule cells, especially the S1 segment, efficiently and effectively reabsorb and transcytose albumin (FIGURE 1) (4, 5). Alterations to albumin such as glycation, carbamylation, and bound ligands affect the renal handling of filtered albumin and likely play a role in PT toxicity and progressive chronic kidney disease (6, 7). Finally, there is increasing use of albumin as a molecular chaperone for specific therapeutics. Therefore, the purpose of this review is to discuss emerging data regarding PT albumin metabolism and provide a framework for considering future studies with direct clinical relevance. Specifically, we outline current data supporting PT reabsorption and transcytosis of filtered albumin and the mechanisms and carriers involved and propose a mechanism for intracellular sorting between degradative and transcytotic pathways based on pH-dependent binding to the neonatal Fc receptor (FcRn).

## 2. PROXIMAL TUBULE

### 2.1. General Function

The PT reabsorbs 65–80% of filtered Na^+^ and H_2_O, all of the amino acids, glucose, 80% of the bicarbonate, and nearly all of the low-molecular-weight proteins (8). The PT consists of three distinct segments, S1, S2, and S3, with cells in each segment having structurally, biochemically, and physiologically distinct characteristics (8–11). TABLE 1 lists key ultrastructural and functional characteristics of the proximal tubule segments.

**Table 1.** Ultrastructural and functional characteristics of proximal tubules

Property	S1	S2	S3
*Ultrastructural*			
Brush border	Rabbit microvilli ∼3 μm	Rabbit microvilli ∼1.6 μmRat shortest microvilli	Rabbit shortest microvilliRat tallest microvilli
	Human similar to rabbit		
Basolateral membrane	Rabbit 16.2 µm^2^/µm^2^	Rabbit 13.4 µm^2^/µm^2^	Rabbit 7.7 µm^2^/µm^2^
	Rat, human, dog, pig similar	Extensive lateral ridges, 2/3 of the cell	Lateral ridges restricted to lower third of the cell
	Extensive lateral ridges >2/3 of cell		
Mitochondria	Most and elongated perpendicular to basement membrane	Less and smaller	Fewest
Lysosomes, vacuoles	Rat large apical vacuoles	Rat smaller and less frequent apical vacuoles	
Endocytic vesicles	Many apical tubules	Many apical tubules	Fewer apical tubules
Other			Lipid droplets
*Functional*			
Fluid uptake, transporters	Dextran uptake	Dextran uptake	
Endocytosis, clathrin mediated and clathrin independent	LMWP (lysozyme) uptake highest		
	Albumin uptake highest		
Megalin	IC-high (rat, rabbit, human)	IC-high (rat, rabbit, human)	IC-lower (rat, rabbit, human)

LMWP, low-molecular-weight protein; IC, immunocytochemistry. See Refs. 12–22.

**FIGURE 2.**
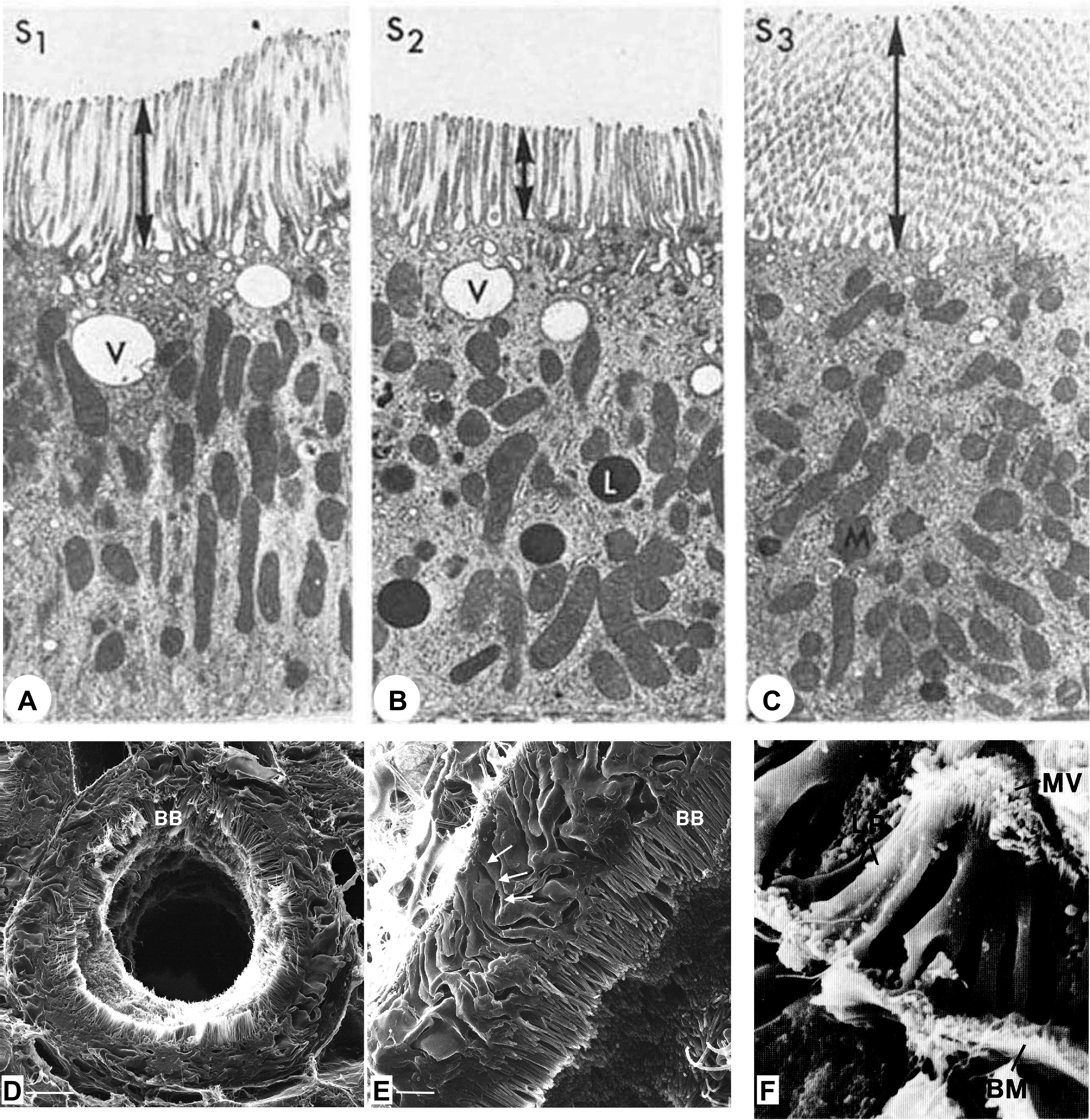
*A–C*: Sprague-Dawley rat kidney proximal tubules (PTs): segment S1 (*A*), S2 (*B*), and S3 (*C*) electron micrographs showing distinct differences in brush border microvilli, mitochondrial organization, and large vesicle/vacuoles between PT segments. L, lysosome; M, mitochondria; V, vacuole. Image from Ref. 22, with permission from *Kidney International*. *D* and *E*: Sprague-Dawley rat PT convoluted tubule at low (*D*; bar, 5 µm) and high (*E*; bar, 1 µm) magnification with helium ion microscopy. Note the bright and prominent brush border (BB) and the complex interdigitations of the lateral cellular membranes of PTs. Image from Ref. 23, with permission from *PLoS One*. *F*: scanning electron micrograph of a rabbit PT showing that lateral ridges (LR) begin below the apical microvilli (MV) and fan laterally. BM, basement membrane.

FIGURE 2 presents conventional electron microscopy images comparing rat PTs (note the presence of vacuoles in S1 and S2, organization of mitochondria, and lysosomes; FIGURE 2, *A–C*) (22) and a low (FIGURE 2*D*)- and a high (FIGURE 2*E*)-magnification image with a high-resolution helium ion scanning microscope that shows the impressive expansion of the rat apical PT membrane due to the extensive brush border (BB) but equally as dramatic the extensive lateral ridges that increase the basolateral membrane (BLM) surface area (23). An earlier study looking at rabbit PTs with scanning electron microscopy (SEM) had also documented this incredible expansion of both apical and basolateral membranes while also highlighting the numerous smaller microvilli located at the base of the lateral ridges (LRs) in contact with the basement membrane (BM) (12). Note that we still do not completely understand how these structural adaptations are utilized by the PTs to perform their varied and important transport functions (13). Differences in ion and glucose transporters, apical and basolateral membrane structures, and endocytic networks have been documented over the last 50 years. Work by Christensen initially characterized the differences in endocytosis between the different rodent PT segments and showed the remarkable rate of endocytosis in vivo (24, 25). The S1 and S2 segments are located in the cortex, and the S3 segment, also known as the straight segment, is in the corticomedullary region. The S1 segment, compared with S2 and S3, has a greater number and greater lengths of apical microvilli, resulting in a larger surface area for reabsorption, and more extensively developed endocytic-lysosomal and Golgi systems. It also has more extensive basolateral interdigitations and a greater number of mitochondria. Therefore, it is not surprising that the S1 segment has the highest capacity for ion, glucose, amino acid, and macromolecule transport of the three segments. The transition from the S1 to the S2 segment is not distinct, but the processes mentioned above are reduced. The change in these cellular processes is substantial as one goes from the S2 to S3 PT cells (9, 20, 26, 27). The different segments also have differing metabolic, autofluorescence, and frequency-domain fluorescence lifetime imaging microscopy signatures allowing for identification in in vivo studies (28, 29). In these studies, the S1 segment, compared with the S2 and S3 segments, was shown to have the greater mitochondrial activity consistent with histological studies. The S1 PT cells extend up into Bowman’s capsule in rodents (30). Finally, as you transition from the cortex into the cortical medullary region there is little structural change in the different proximal tubule segments arising from the glomerulus.

### 2.2. Endocytosis by the Proximal Tubule

Proximal tubule reabsorption and metabolism of macromolecules was initially characterized by micropuncture, microperfusion, autoradiographic, histological, and electron microscopy (EM) techniques using tracers (31, 32). Classically, cellular uptake, processing, and transcytosis of proteins and other molecules by endocytotic pathways has been attributed primarily to clathrin-mediated endocytosis (CME) by apical membrane-bound receptors clustering into clathrin-coated pits. FIGURE 1 provides an overview of filtration, reabsorption via endocytosis, and intracellular processing with either degradation within lysosomes or transcytosis across the basolateral membrane. Depending upon the cell type, coated pits can make up between 0.4% and 3.8% of the cell’s surface (33). Numerous reviews cover this topic (34, 35). Fluid-phase endocytosis (FPE) and caveolin-dependent internalization also internalize proteins, but caveolin-dependent processes do not exist in PT. These processes, while not being specific, are directed along the sorting endosomal compartment to undergo either degradation through lysosomal pathways or transcytosis based on FcRn binding (36). Fluid-phase endocytosis is a quantitatively important process in PTs as shown by the rapid and extensive non-receptor-mediated apical uptake of neutral fluorescent dextrans, markers of fluid-phase endocytosis (20, 37, 38). Note that FPE comprises multiple types of clathrin-independent endocytosis mechanisms including fast endophilin-mediated endocytosis (FEME), clathrin-independent carrier/glycosylphosphatidylinositol (GPI)-anchored protein-enriched early endosomal compartments (CLIC-GECs), and massive endocytosis (MEND), which are discussed in sect. 3.2 (see FIGURE 5).

All segments of the PT endocytose fluid and filtered glomerular ligands, with a gradient existing between S1–S3 segments. Clathrin-coated pits and the resulting internalized endocytic vesicles are most extensive in S1, less in S2, and far less to nonexistent in the S3 segment (9, 20, 39). Endocytic activity at the brush border is a highly active process, with the amount of surface membrane contained in apical membrane invaginations internalized every 78 s (25). This rapid endocytic rate reabsorbs luminal fluid and may play a role in increased PT reabsorption in response to volume depletion. However, differentiating CME and FPE, and quantifying the overall importance of fluid-phase endocytosis, is challenging, as all endocytic vesicles contain fluid and thus luminal contents. Therefore, CME vesicles also contain fluid-phase markers, i.e., dextrans and FPE vesicles also contain receptor-mediated markers, respectively. Mouse transgenic and knockout (KO) studies of CME receptors are discussed below and have provided significant insight.

**FIGURE 3.**
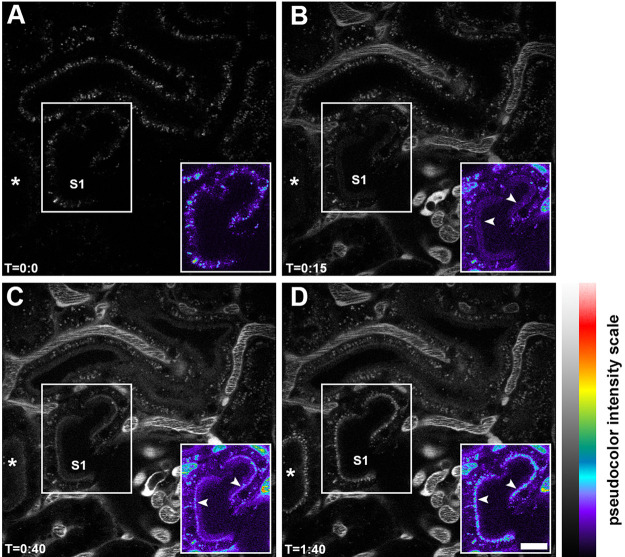
Initial filtration, binding, and internalization of fluorescent albumin by proximal tubule cells. An intravital 2-photon image of an S1 proximal tubule section is shown before an infusion of Texas Red-X-rat serum albumin (TR-RSA) in *A*. The adjacent tubule images (asterisks) are a continuation of the same S1 segment. The *insets* at *bottom right* in all panels show the S1 segment in a pseudocolor palette to better discern dimmer intensities not readily evident in black-and-white version. The micrograph in *B* was taken 15 s after the initial infusion. A portion of the glomerulus associated with the S1 segment is under the pseudocolor *inset*. The *inset* in *B* demonstrates early binding at the apical brush border membrane (arrowheads in *inset*). Early brush border binding progresses and eventually enriches in the subapical region of the S1 segment, appearing in *C* as a distinct band (arrows in *inset*). The end of the 100-s movie (*D*; Supplemental Movie 1) clearly shows small, distinct, early endocytic vesicles lining the subapical region, with a few appearing to have traversed well into the cytosol of the tubular epithelia. The individual time stamps are located at *bottom left* of all panels. In Supplemental Movie 1, the vascular intensity of albumin can be seen fluctuating in the earlier portion. This is due to the careful and protracted bolus infusion of TR-RSA in an effort to avoid saturation of fluorescence in the plasma. The bar located on *right* of *D* shows intensity equivalence between the black-and-white and pseudocolor display palettes. Bar, 20 µm. Data from Ref. 12.

**FIGURE 4.**
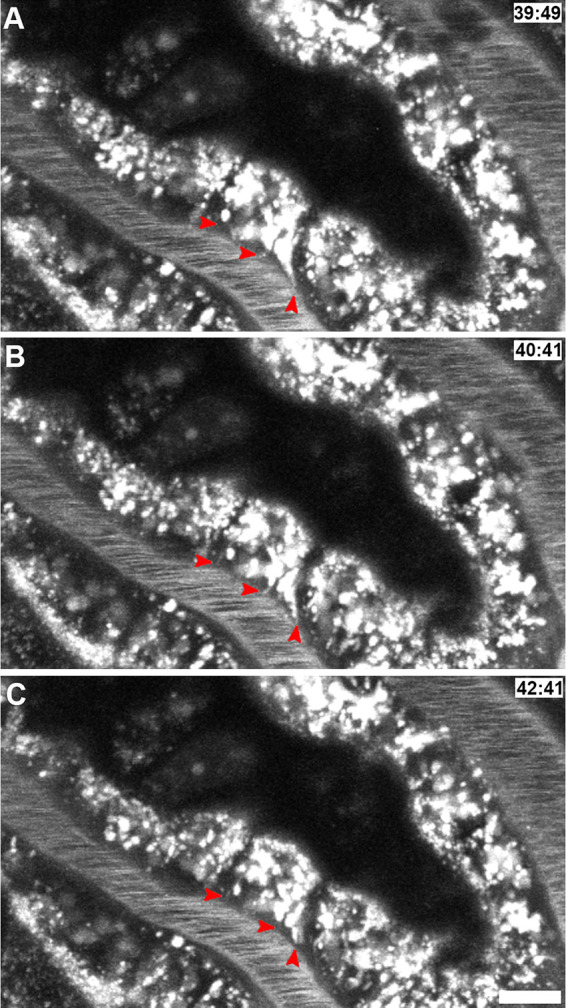
Intracellular trafficking of albumin in proximal tubule cells. A high-resolution, 100-frame, 5-µm, 4-dimensional volume of Texas Red-X-albumin (TR-RSA) trafficking within rat renal proximal tubule cells shows vesicular and tubular-vesicular trafficking. Three micrographs from the data are shown in *A–C*, with respective time stamps from initial infusion of the fluorescent albumin. The data show small endocytic vesicles readily moving on the luminal side of the proximal tubule (lumen) shuttling around the apical region. These vesicles can also be seen moving toward the basolateral membrane, adjacent to the microvasculature showing rapidly flowing red blood cells. Arrowheads point to bright accumulations of the TR-RSA (with a dye-to-protein ratio of 1:1), showing distinct tubular-vesicular extensions projecting toward the basolateral membrane and appearing to merge with the interstitial space. Arrowheads point to regions where prominent extensions form and shuttle larger, brighter vesicles along these dimmer albumin-containing tracts. The often subtle fluorescence of these structures necessitated acquisition of these images with some degree of saturation in the brighter regions to allow for detection of the dimmer structures. The same structures can be seen in other cells throughout the proximal tubules shown here. Bar, 10 µm. Image from Ref. 4 (Supplemental Movie 2), with permission from the *Journal of the American Society of Nephrology*.

Intravital two-photon microscopy techniques have enabled rapid dynamic intracellular processes of PTs to be observed and quantified in the kidney (40–46). This is well demonstrated for albumin (FIGURES 3 AND 4, and Supplemental Movies 1 and 2, available at https://doi.org/10.6084/m9.figshare.14665968) (4). This technique has allowed increased understanding of proximal tubule cell (PTC) structure-function relationships, cell-cell interactions, multiple cellular events occurring simultaneously, and interactions of PTs with the vasculature and blood cells, glomerular filtrate, and the interstitium (47–49).

Multiphoton microscopy does have limitations that have been previously reviewed (50). These include limited depth of penetration to ∼100 µm with reasonable resolution. This has limited mouse studies, in particular, as no glomeruli lie within reach of multiphoton microscopy after ∼4 wk of age without manipulations such as prolonged ureteral ligation. Many of these limitations, particularly sample stability, have been characterized and addressed over many years of utilizing multiphoton microscopy to study the intact kidney. A challenging factor that remains constant is that of phototoxicity and photobleaching, which increases with moderate increases in multiphoton illumination. However, advancements in photodetector sensitivity have allowed the use of lower laser power transmissivity, thus reducing phototoxicity/photobleaching. Moreover, the development of new fluorescent compounds, which have increased quantum efficiency and do not impact the experimental system, has contributed to improving signal to noise while minimizing the laser excitation power required. These and future advances will all continue to contribute to more information being collected at each time point, while limiting photobleaching and/or toxicity.

Proximal tubule cell endocytosis has been well characterized and shown to be rapid, dynamic, and regulated (3, 4, 43–45). Quantitation of cortical PT endocytosis is possible for both S1 and a combination of S1 and S2 segments, using multiple markers with variable molecular weights, charge, and fluorophores (42). Marked axial differences in ligand uptake and endocytic function have been shown to exist along the PT and to be independent of megalin expression. Using low-molecular-weight proteins (LMWPs) and dextrans labeled with different fluorophores and tissue clearing techniques, Schuh et al. were able to characterize differences in CME and FPE within the different PT segments. Although the S1 segment had both CME and FPE, S2–S3 only had FPE as determined by dextran uptake. Megalin distribution was similar in the three segments (9, 20). The PT segmental distribution of cubilin was highest in S1 and decreased to a similar extent in S2–S3. Disabled Homolog 2 (Dab2) is an adapter protein that functions as a clathrin-associated sorting protein (CLASP) required for clathrin-mediated endocytosis of selected cargo proteins. It is predominantly located in S1 and decreases in a stepwise fashion going from S1 to S2 to S3 (9, 20). Interestingly, with induction of increased glomerular proteinuria the S3 segment became more like the S2 segment in transporting LMWP and Dab2 increased in concert (9). Thus, there seems to be plasticity in the S3 segment in response to increased delivery of protein within the filtrate.

Little is known about the regulation of endocytosis in PTs. However, examples of regulation include the ability of the PT to respond to high serum protein levels with a reduction in reabsorption of filtered albumin leading to increased albuminuria (3). Proximal tubules can also selectively stop aminoglycoside reabsorption during long-term exposure to aminoglycosides (51, 52). Furthermore, in a series of manuscripts the Weisz laboratory (53–55) delineated the mechanism of fluid shear stress-induced apical endocytosis via a mechanosensation of the primary cilia requiring extracellular Ca^2+^, release of cellular ATP, and stimulation of purinergic receptor (P2R) (56). Cultured proximal tubule cells responded to fluid sheer stress via mammalian target of rapamycin (mTOR) and upregulated endocytic capacity, mitochondrial function, and lysosome biogenesis (57). Long-term inhibition of mTOR in mice caused a reduction in apical megalin and was associated with proteinuria (53). Finally, a conditional knockout of mTOR complex (mTORC)1 and mTORC2 subunits in mouse proximal tubule resulted in impaired endocytic capacity due to severely reduced apical microvilli and decreased clathrin-coated pits (58).

Highly active endocytic cells like PTCs use lysosomes as key degradative compartments and as a signaling hub involved in nutrient sensing through its dynamic association with mTORC1 (59–61). In addition, recent identification of lysosome-mitochondria membrane contact sites and regulation by Rab7 suggest that these organelles have significant cross talk, with both being critical for proper metabolism and degradation (62). Furthermore, disruption of lysosome pH has been shown to decrease mitochondrial respiration (63, 64). Consequently, their in vivo dynamic changes are being actively investigated for their roles in kidney disease and cellular homeostasis (65–67).

## 3. ENDOCYTOSIS—TYPES, MECHANISMS, AND PRINCIPAL COMPONENTS

### 3.1. Clathrin-Mediated Endocytosis

**FIGURE 5.**
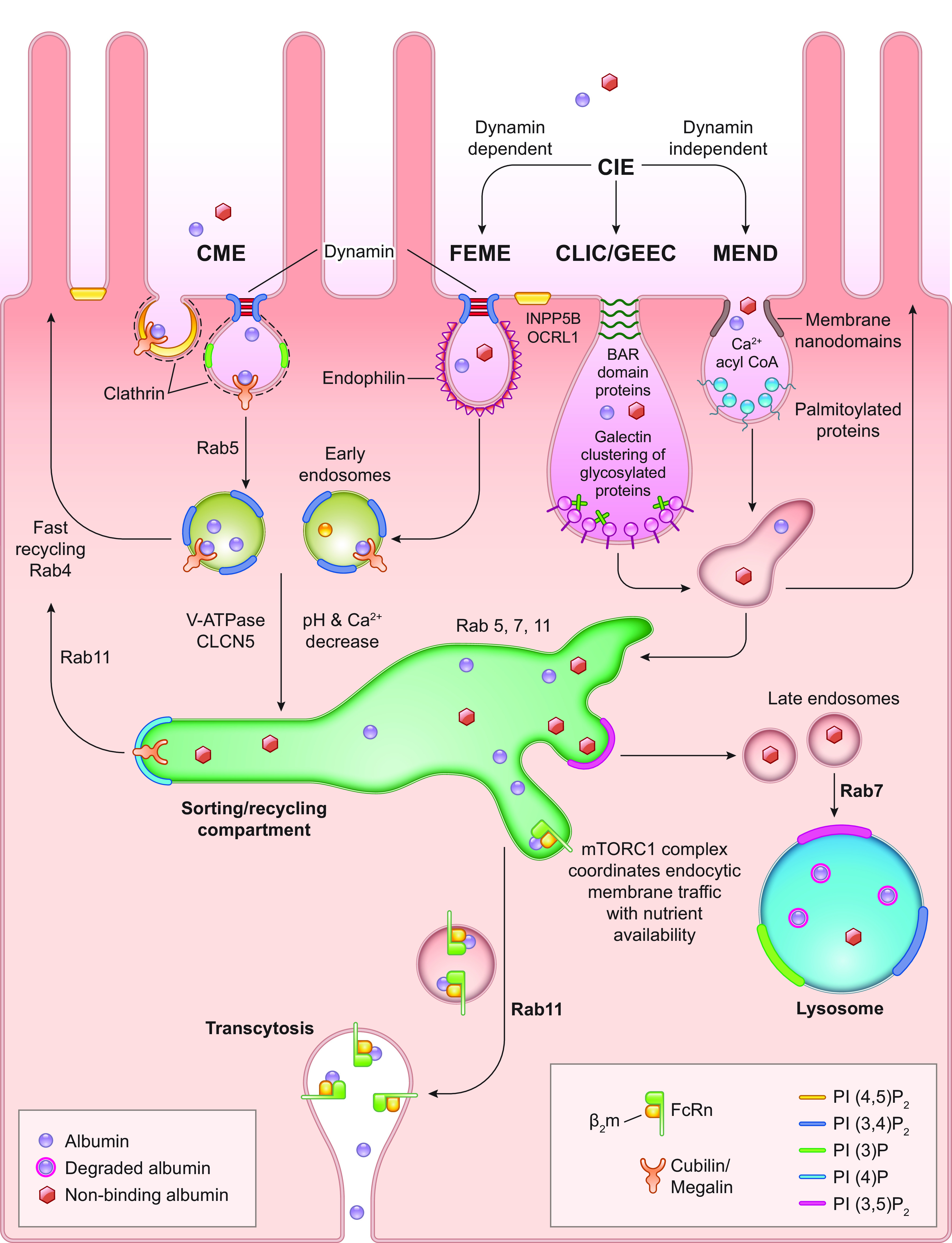
Albumin reabsorption and trafficking by proximal tubule cells. Albumin reabsorbed by clathrin-mediated endocytosis (CME) or clathrin-independent endocytosis (CIE) undergoes endosomal acidification, resulting in dissociation of albumin from megalin-cubilin complexes for CME endosomes. Albumin binding to neonatal Fc receptor (FcRn) will occur as pH decreases, with a possible role for Ca^2+^ decrease. This transfer occurs in the dynamic sorting/recycling compartment. This exchange within the sorting compartment directs albumin either toward lysosomal degradation or to the transcytotic pathway. Both vesicular and tubular structures mediate albumin transcytosis to the basolateral membrane. Vesicle fusion with the basolateral membrane exposes its contents to the interstitial fluid, at elevated pH, resulting in dissociation of albumin from FcRn. FcRn undergoes recycling back to the sorting compartment. Reductions in albumin-FcRn binding within the endosomal compartment by albumin alterations such as oxidation, glycosylation, or carbamylation (nonbinding albumin) would reduce transcytosis of albumin. This provides an intracellular molecular sorting mechanism preserving physiological albumin and facilitating catabolism of albumin not binding to FcRn. It could also result in catabolism of albumin if concentrations exceed FcRn binding capacity. Note that multiple genetic mutations, knockouts, and specific manipulations to proteins involved in these intricate traffic and sorting pathways, i.e., Rabs, phosphatidylinositol (PI) kinase, phosphatases, V-ATPase, CLCN5, and mammalian target of rapamycin complex (mTORC)1 can lead to dysfunction. CLIC/GEEC, clathrin-independent carrier/glycosylphosphatidylinositol (GPI)-anchored protein-enriched early endosomal compartments; FEME, fast endophilin-mediated endocytosis; MEND, massive endocytosis; PI(3)P, phosphatidylinositol 3-phosphate; PI(4)P, phosphatidylinositol 4-phosphate; PI(3,4)P_2_, phosphatidylinositol 3,4-bisphosphate; PI(3,5)P_2_, phosphatidylinositol 3,4-bisphosphate; PI(4,5)P_2_, phosphatidylinositol 4,5-bisphosphate; β_2_m, β2-microglobulin.

Clathrin-mediated endocytosis (CME) has been studied for almost 50 years after the initial identification of clathrin in 1975 (68). Many excellent reviews have been written describing the present knowledge of the mechanism, regulation, biochemistry, and interacting molecules that when combined reveal a very dynamic and complex process (68–71) (FIGURE 5). However, it is important to note that many of these data were obtained in cell culture models that may not replicate the in vivo mechanism(s) (72). In fact, a recent paper compared the transcriptomes of multiple proximal tubule cell lines, including MDCK, LLC-PK1, and OK, to isolated mouse PTs, with the highest percentage match being only 45% of proximal marker genes (73). Our focus is on highlighting the evidence for albumin CME, with an emphasis on presenting data from in vivo studies and polarized epithelial cell culture models. TABLE 2 lists major components, i.e., clathrin, cubilin, megalin, etc., critical for endocytic processes, their human UniProtKB ID and Function, mass, mRNA and protein amount determined from isolated rat PT tubule segments, and mRNA from isolated mouse PT tubule segments (74–76). Additional targeted -omics research is needed to compare and evaluate what genes and proteins are present in each tubule segment and localized to specialized cellular domains from different species (77). Although early studies revealed many key differences, the technology, in particular mass spectrometry, has improved dramatically over the last 20 years and more targeted -omics approaches are definitely warranted (77).

**Table 2.** Major components for endocytic processes

Protein	HUMAN UniProtKB ID and Function (https://www.uniprot.org/)	Mass, Da	Rat PT Segment Values (S1-S2-S3)		
			Rat RNA-Seq Analysis (74)	Rat Proteomic Analysis (75)	Mouse RNA-Seq Analysis (76)
Clathrin heavy chain 1 (CLTC)	Q00610. Clathrin is the major protein of the polyhedral coat of coated pits and vesicles. Two different adapter protein complexes link the clathrin lattice either to the plasma membrane or to the *trans*-Golgi network.	191,615	24-9-36	1353031-3459647-2380936	81-67-42
Megalin (LRP2)	P98164. Multiligand endocytic receptor	521,958	19-34-15	1526800-3194703-1744919	106-84-34
Disabled homolog 2 (DAB2)	P98082. Adapter protein that functions as clathrin-associated sorting protein (CLASP) required for clathrin-mediated endocytosis of selected cargo proteins. Can bind and assemble clathrin and binds simultaneously to phosphatidylinositol 4,5-bisphosphate [PtdIns(4,5)P_2_] and cargos containing nonphosphorylated NPXY internalization motifs, such as the LDL receptor, to recruit them to clathrin-coated pits. Can function in clathrin-mediated endocytosis independently of the AP-2 complex.	82,448	63-67-3	4315208-4301587-569388	424-189-126
Cubilin (CUBN)	O60494. Endocytic receptor that plays a role in lipoprotein, vitamin, and iron metabolism by facilitating their uptake. Acts together with LRP2 to mediate endocytosis of high-density lipoproteins, GC, hemoglobin, ALB, TF, and SCGB1A1. Acts together with AMN to mediate endocytosis of the CBLIF-cobalamin complex.	398,736	9-8-9	679335-419164-150646	26-24-19
Amnionless (AMN)	Q9BXJ7. Membrane-bound component of the endocytic receptor formed by AMN and CUBN. Required for normal CUBN glycosylation and trafficking to the cell surface. The complex formed by AMN and CUBN is required for efficient absorption of vitamin B_12_.	47,754	12-10-4	220780-113813-35082	292-400-151
Low-density lipoprotein (LDL) receptor-related protein-associated protein (LRPAP1), (RAP)	P30533. Molecular chaperone for LDL receptor (LDLR)-related proteins that may regulate their ligand binding activity along the secretory pathway.	41,466	107-133-172	2368015-5247423-3376969	624-1570-1181
LRP chaperone MESD (MESD)	Q14696. Chaperone specifically assisting the folding of beta-propeller/EGF modules within the family of LDLRs.	26,077	6.7-4.3-1.5	453096-921328-1349473	24-21-20
Unconventional myosin-VI (MYO6)	Q9UM54. Unconventional myosins serve in intracellular movements (by similarity). Myosin 6 is a reverse-direction motor protein that moves toward the minus end of actin filaments. Appears to be involved in a very early step of clathrin-mediated endocytosis in polarized epithelial cells. May play a role in transporting DAB2 from the plasma membrane to specific cellular targets.	149,691	0-0-0	538230-2033927-1268191	86-116-53
Low-density lipoprotein receptor adapter protein 1(LDLRAP1 or ARH)	Q5SW96. Adapter protein [clathrin-associated sorting protein (CLASP)] required for efficient endocytosis of the LDLR in polarized cells such as hepatocytes and lymphocytes but not in nonpolarized cells (fibroblasts).	33,885	0-3-0	9462-771-27874	8.7–9.3-6.2
Nuclear valosin-containing protein-like (NVL)	O15381. Participates in the assembly of the telomerase holoenzyme and effecting of telomerase activity via its interaction with TERT. Involved in both early and late stages of the pre-rRNA processing pathways.	95,051	0-0.1-0	0-7-0	4.8–5.2-3.4
Calnexin (CANX)	P27824. Calcium-binding protein that interacts with newly synthesized glycoproteins in the endoplasmic reticulum (ER). It may act in assisting protein assembly and/or in the retention within the ER of unassembled protein subunits. It seems to play a major role in the quality control apparatus of the ER by the retention of incorrectly folded proteins.	67,568	34-35-10	463935-1456069-1435379	217-204-88
Calreticulin (CALR)	P27797. Calcium-binding chaperone that promotes folding, oligomeric assembly, and quality control in the ER via the calreticulin/calnexin cycle. This lectin interacts transiently with almost all of the monoglucosylated glycoproteins that are synthesized in the ER.	48,142	14-41-36	2504760-4891714-7992581	430-496-336
UDP-glucose: glycoprotein glucosyl-transferase 1 (UGGT1)	Q9NYU2. Recognizes glycoproteins with minor folding defects. Reglucosylates single *N*-glycans near the misfolded part of the protein, thus providing quality control for protein folding in the ER. Reglucosylated proteins are recognized by calreticulin for recycling to the ER and refolding or degradation.	177,190	0.7–2.5-3	290005-126043-59116	17-14-6.0
Phosphatidylinositol-binding clathrin assembly protein (PICALM)	Q13492. Cytoplasmic adapter protein that plays a critical role in clathrin-mediated endocytosis, which is important in processes such as internalization of cell receptors, synaptic transmission, or removal of apoptotic cells. Recruits adaptor protein complex 2 (AP-2) and attaches clathrin triskelions to the cytoplasmic side of plasma membrane, leading to clathrin-coated vesicle (CCV) assembly.	70,755	10-26-32	361164-1005072-596570	39-17-27
AP-2 complex subunit beta (AP2B1)	P63010. Component of the adaptor protein complex 2 (AP-2). Adaptor protein complexes function in protein transport via transport vesicles in different membrane traffic pathways. Adaptor protein complexes are vesicle coat components and appear to be involved in cargo selection and vesicle formation. AP-2 is involved in clathrin-dependent endocytosis in which cargo proteins are incorporated into vesicles surrounded by clathrin (CCVs), which are destined for fusion with the early endosome.	104,553	0-0.1-0	364777-533130-344635	9.7-7.8-5.9
AP-2 complex subunit alpha-2 (AP2A2)	O94973. Component of the AP-2	103,960	9.4-33-23	366498-882680-341876	107-88-46
AP-2 complex subunit mu (AP2M1)	Q96CW1. Component of the AP-2	49,655	69-192-66	414573-370192-121823	140-140-95
Dynamin-1-like protein (Dnm1l)	O00429. Required for formation of endocytic vesicles	81,877	0.1–3.5-0.1	39030-124829-134402	17-18-19
Dynamin-2 (Dnm2)	P50570. Plays an important role in vesicular trafficking processes, in particular endocytosis.	98,064	4-8.7-8.7	114242-155062-139558	48-84-48
Dynamin-3 (Dnm3)	Q9UQ16, Microtubule-associated force-producing protein involved in producing microtubule bundles and able to bind and hydrolyze GTP. Most probably involved in vesicular trafficking processes, in particular endocytosis (by similarity).	97,746	0-0-0	0-6653-0	1.5-0.3-0.6
Phosphatidylinositol 4-phosphate 3-kinase C2 domain-containing subunit alpha (PIK3C2A)	O00443. Generates phosphatidylinositol 3-phosphate (PtdIns3P) and phosphatidylinositol 3,4-bisphosphate [PtdIns(3,4)P_2_], which act as second messengers. Has a role in several intracellular trafficking events. Functions in clathrin-coated endocytic vesicle formation and distribution. Regulates dynamin-independent endocytosis, probably by recruiting EEA1 to internalizing vesicles.	190,680	0-0-0	1934-7283-12	19-11-8
Sorting nexin-9 (SNX9)	Q9Y5X1. Plays a role in endocytosis via clathrin-coated pits but also clathrin-independent, actin-dependent fluid-phase endocytosis. Plays a role in macropinocytosis. Stimulates the GTPase activity of DNM1. Promotes DNM1 oligomerization. Promotes activation of the Arp2/3 complex by WASL and thereby plays a role in the reorganization of the F-actin cytoskeleton. Binds to membranes enriched in PtdIns(4,5)P_2_ and promotes membrane tubulation. Has lower affinity for membranes enriched in PtdIns3P.	66,592	1.4–1.6-0.8	8563-206035-26563	52-60-26
Sorting nexin-12 (SnX12)	Q9UMY4. May be involved in several stages of intracellular trafficking.	18,885	0-0-0	950269-284005-199264	30-19-19
Heat shock cognate 71-kDa protein (HSPA8)	P11142. Molecular chaperone implicated in a wide variety of cellular processes, including protection of the proteome from stress, folding, and transport of newly synthesized polypeptides, activation of proteolysis of misfolded proteins, and the formation and dissociation of protein complexes.	70,898	0.1-0-0.3	10799513-25268973-25522023	774-900-494
Ras-related protein Rab-4A (RAB4A)	P20338. Small GTPase that cycles between an active GTP-bound and an inactive GDP-bound state, involved in protein transport.	24,390	2.4-0.3–0.4	22562-25590-395	91-65-71
Ras-related protein Rab-4B (RAB4B)	P61018. Small GTPase that cycles between an active GTP-bound and an inactive GDP-bound state, involved in protein transport.	23,587	0.1–0.5-1.9	288-0-0	38-49-45
Ras-related protein Rab-5A (RAB5A)	P20339. RAB5A is required for the fusion of plasma membranes and early endosomes.	23,659	0.3–0.4-0	0-104769-384040	42-76-58
Ras-related protein Rab-5B (RAB5B)	P61020. Protein transport. Probably involved in vesicular traffic.	23,707	6-5.5–5.9	289717-77304-2070	10-11-12
Ras-related protein Rab-5C (RAB5C)	P51148. Protein transport. Probably involved in vesicular traffic.	23,483	3.6–13.1-8.9	892951-2135297-1481347	59-12-16
Ras-related protein Rab-7A (RAB7A)	P51149. Small GTPase that cycles between active GTP-bound and inactive GDP-bound states. In its active state binds to a variety of effector proteins playing a key role in the regulation of endo-lysosomal trafficking. Governs early-to-late endosomal maturation, microtubule minus end as well as plus end-directed endosomal migration and positioning, and endosome-lysosome transport through different protein-protein interaction cascades.	23,490	23-19-48	282332-1789658-2848084	Rab7-249-216-150
Ras-related protein Rab-11A (RAB11A)	P62491. The small Rab GTPase RAB11A regulates endocytic recycling.	24,394	48-27-6	0-0-0	317-217-196
Ras-related protein Rab-11b (RAB11B)	Q15907. The small Rab GTPase RAB11B plays a role in endocytic recycling, regulating apical recycling of several transmembrane proteins including cystic fibrosis transmembrane conductance regulator/CFTR, epithelial sodium channel/ENaC, potassium voltage-gated channel, and voltage-dependent L-type calcium channel. May also regulate constitutive and regulated secretion, like insulin granule exocytosis.	24,489	22-18-35	790325-2821196-3176606	49-39-36
Ras-related protein Rab-7L1 (RAB29)	O14966. The small GTPases Rab are key regulators in vesicle trafficking. Essential for maintaining the integrity of the endosome-*trans*-Golgi network structure (by similarity).	23,155	19-48-79	0-24940-29866	24-30-30
Rab11 family-interacting protein 3(RAB11FIP3)	O75154. Acts as a regulator of endocytic traffic by participating in membrane delivery. Acts as an adapter protein linking the dynein motor complex to various cargos and converts dynein from a nonprocessive to a highly processive motor in the presence of dynactin. Facilitates the interaction between dynein and dynactin and activates dynein processivity (the ability to move along a microtubule for a long distance without falling off the track).	82,440		228223-210708-93172	394-311-197
Early endosome antigen 1 (EEA1)	Q15075. Binds phospholipid vesicles containing PtdIns3P and participates in endosomal trafficking.	162,466	0-0-8	146701-248972-245684	15-16-12
Rab-interacting lysosomal protein (RILP)	Q96NA2. Rab effector playing a role in late endocytic transport to degradative compartments. Involved in the regulation of lysosomal morphology and distribution. Induces recruitment of dynein-dynactin motor complexes to Rab7A-containing late endosome and lysosome compartments.	44,200	4.5-0.4-0	659-24930-19	14-14-9
Phosphatidylinositol 3-kinase catalytic subunit 3 (PIK3C3, hVPS34)	Q8NEB9. Catalytic subunit of the PI3K complex that mediates formation of phosphatidylinositol 3-phosphate; different complex forms are believed to play a role in multiple membrane trafficking pathways: PI3KC3-C1 is involved in initiation of autophagosomes and PI3KC3-C2 in maturation of autophagosomes and endocytosis. As part of PI3KC3-C1, promotes ER membrane curvature formation prior to vesicle budding.	101,549	0.1-0-0.2	0-4345-0	11-13-8
Vacuolar fusion protein MON1 homolog A (MON1A)	Q86VX9. Plays an important role in membrane trafficking through the secretory apparatus. Not involved in endocytic trafficking to lysosomes (by similarity). Acts in concert with CCZ1, as a guanine exchange factor (GEF) for RAB7, promotes the exchange of GDP to GTP, converting it from an inactive GDP-bound form into an active GTP-bound form.	72,895	2.4–13.5-4.4	0-7.1-379	12-23-13
Guanine nucleotide exchange factor for Rab-3A (RAB3IL1)	Q8TBN0. Guanine nucleotide exchange factor (GEF) that may activate RAB3A, a GTPase that regulates synaptic vesicle exocytosis. Promotes the exchange of GDP to GTP, converting inactive GDP-bound Rab proteins into their active GTP-bound form. May also activate RAB8A and RAB8B.	42,637	2.4-0.1-0	23143-0-0	151-22-14
Ras-related protein Rab-8A (RAB8A)	P61006. The small GTPases Rab are key regulators of intracellular membrane trafficking, from the formation of transport vesicles to their fusion with membranes.	23,668	10.9-8.3-23.2	106639-360391-227965	64-103-90
Flotillin-1 (FLOT1)	O75955. May act as a scaffolding protein within caveolar membranes, functionally participating in formation of caveolae or caveolae-like vesicles.	47,355	0-0-0	26432-88562-94444	26-36-39
Sodium/hydrogen exchanger 3 (SLC9A3)	P48764. Involved in pH regulation to eliminate acids generated by active metabolism or to counter adverse environmental conditions. Major proton extruding system driven by the inward sodium ion chemical gradient, NHE3.	92,855	0.5–4.1-0	10807-105835-0	37-15-0
Na^+^/H^+^ exchange regulatory cofactor NHE-RF1 (SLC9A3R1)	O14745. Scaffold protein that connects plasma membrane proteins with members of the ezrin/moesin/radixin family and thereby helps to link them to the actin cytoskeleton and to regulate their surface expression. Necessary for recycling of internalized ADRB2. Was first known to play a role in the regulation of the activity and subcellular location of SLC9A3. Involved in the regulation of phosphate reabsorption in the renal proximal tubules.	38,868	277-200-329	7459505-13411180-37409278	215-458-335
H^+^/Cl^−^ exchange transporter 5 (CLCN5)	P51795. Proton-coupled chloride transporter. Functions as antiport system and exchanges chloride ions against protons. Important for normal acidification of the endosome lumen.	90,785	0-0.1-0	0-440-567	12-13-4
V-type proton ATPase subunit C 1 (ATP6V1C1)	P21283. Subunit of the peripheral V1 complex of vacuolar ATPase. Subunit C is necessary for the assembly of the catalytic sector of the enzyme and is likely to have a specific function in its catalytic activity. V-ATPase is responsible for acidifying a variety of intracellular compartments in eukaryotic cells.	43,942	4.8-2.1-0.3	704926-545040-225751	146-95-85
Endophilin-A2 (SH3GL1)	Q99961. Implicated in endocytosis. May recruit other proteins to membranes with high curvature (by similarity).	41,490	0.1–1.7-0.1	7416-460-51270	26-34-32
Endophilin-B1 (SH3GLB1)	Q9Y371. May be required for normal outer mitochondrial membrane dynamics. Required for coatomer-mediated retrograde transport in certain cells (by similarity). May recruit other proteins to membranes with high curvature. May promote membrane fusion.	40,796	10-4-7	20735-82723-151211	38-92-68
Endophilin-B2 (SH3GLB2)	Q9NR46	43,974	3.4–5.6-1.2	0-9574-6245	20-32-33
Type II inositol 1,4,5-trisphosphate 5-phosphatase (INPP5B)	P32019. Hydrolyzes PtIns(4,5)P_2_ and the signaling molecule phosphatidylinositol 1,4,5-trisphosphate [PtIns(1,4,5)P_3_], and thereby modulates cellular signaling events. The inositol polyphosphate 5-phosphatase INPP5B is a gene paralog of the Lowe syndrome OCRL1, sharing similar substrate specificity, domain organization, and an ability to partially compensate for loss of OCRL1 in knockout mice.	112,852	0-0-0	6059-4252-0	47-71-42
Inositol polyphosphate 5-phosphatase K (INPP5K)	Q9BT40. Inositol 5-phosphatase that acts on inositol 1,4,5-trisphosphate, inositol 1,3,4,5-tetrakisphosphate, phosphatidylinositol 4,5-bisphosphate, and phosphatidylinositol 3,4,5-trisphosphate.	51,090	3-16-6	310-20976**-**14332	29-41-28
Inositol polyphosphate 5-phosphatase OCRL (OCRL)	Q01968. Catalyzes the hydrolysis of the 4-position phosphate of PtdIns(4,5)P_2_ and phosphatidylinositol-3,4,5-bisphosphate [PtdIns(3,4,5)P_3_], with the greatest catalytic activity toward PtdIns(4,5)P_2_. Regulates traffic in the endosomal pathway by regulating the specific pool of phosphatidylinositol 4,5-bisphosphate that is associated with endosomes.	104,205	0-0-0	0-0-0	4.7-3.9-1.6
Cell division control protein 42 homolog (Cdc42)	Q8CFN2. Plasma membrane-associated small GTPase that cycles between an active GTP-bound and an inactive GDP-bound state. In active state binds to a variety of effector proteins to regulate cellular responses. Involved in epithelial cell polarization processes.	21,259	99-43-35	1483102-1501156-1234699	274-176-168
Galectin-2 (LGALS2)	P05162. This protein binds beta-galactoside. Its physiological function is not yet known.	14,644	2.2-2.2-0.3	105420-998-0	0-0-0
Pantothenate kinase 2, mitochondrial (PANK2)	Q9BZ23. Catalyzes the phosphorylation of pantothenate to generate 4′-phosphopantothenate in the first and rate-determining step of coenzyme A (CoA) synthesis.	62,681	0-0.1-0.17656-0-017-37-23		
IgG receptor FcRn large subunit p51 (FCGRT)	P55899. Cell surface receptor that transfers passive humoral immunity from the mother to the newborn. Binds to the Fc region of monomeric immunoglobulin gamma and mediates its selective uptake from milk. Mechanistically, monomeric IgG binding to FcRn in acidic endosomes of endothelial and hematopoietic cells recycles IgG to the cell surface, where it is released into the circulation. In addition of IgG, regulates homeostasis of the other most abundant circulating protein albumin/ALB.	39,743	5-9-23	0-0-0	387-503-274
Beta-2-microglobulin (B2M)	P61769, Component of the class I major histocompatibility complex (MHC).	13,715	457-159-165	274038-256333-146232	633-129-142
Vesicle-associated membrane protein 8 (VAMP8)	Q9BV40. Soluble *N*-ethylmaleimide-sensitive factor-attachment protein receptors (SNAREs) are essential proteins for fusion of cellular membranes. SNAREs localized on opposing membranes assemble to form a trans-SNARE complex, an extended, parallel 4 alpha-helical bundle that drives membrane fusion. VAMP8 is a SNARE involved in autophagy through the direct control of autophagosome membrane fusion with the lysosome membrane via its interaction with the STX17-SNAP29 binary t-SNARE complex. Involved in the homotypic fusion of early and late endosomes (by similarity).	11,438	150-166-409	1866526-880126-427091	405-595-1149
Rab11 family-interacting protein 5 (RAB11FIP5)	Q9BXF6. Rab effector involved in protein trafficking from apical recycling endosomes to the apical plasma membrane. Involved in insulin granule exocytosis. May regulate V-ATPase intracellular transport in response to extracellular acidosis	70,415		319299-84569-1139	18-10-8
Serine/threonine-protein kinase mTOR (MTOR)	P42345. Serine/threonine protein kinase that is a central regulator of cellular metabolism, growth, and survival in response to hormones, growth factors, nutrients, energy, and stress signals.	288,892	0.4-0.3-0	2358-9864-9188	15-115-115
Regulatory-associated protein of mTOR (RPTOR)	Q8N122. Involved in the control of the mammalian target of rapamycin complex 1 (mTORC1) activity, which regulates cell growth and survival and autophagy in response to nutrient and hormonal signals; functions as a scaffold for recruiting mTORC1 substrates.	149,038	0.4-0-0	0-2695-0	3.9–5.7-5.1
Rapamycin-insensitive companion of mTOR (RICTOR)	Q6R327. Subunit of mTORC2, which regulates cell growth and survival in response to hormonal signals. mTORC2 is activated by growth factors but, in contrast to mTORC1, seems to be nutrient insensitive. mTORC2 seems to function upstream of Rho GTPases to regulate the actin cytoskeleton, probably by activating one or more Rho-type guanine nucleotide exchange factors.	192,218		0-9.6-0	10-6.3–9.5
Sodium/glucose cotransporter 2 (SLC5A2, SGLT2)	P31639. Sodium-dependent glucose transporter. Has a Na^+^-to-glucose coupling ratio of 1:1. Mutations result in renal glucosuria and inhibitors improve kidney and cardiac outcomes including eGFR and albuminuria levels.	72,897	91-8.2-0.5	2452145-53473-544	2722-0.7-0
Ras-related protein Rab-38 (RAB38)	P57729. Data suggest that Rab38 affects urinary protein excretion via effects in the proximal tubule.	23,712	0-0-0	0-0-0	2.2-32-49

EGF, epidermal growth factor; RNA-seq, RNA sequencing; GC, group specific component; ALB, albumin; TF, transferrin; eGFR, estimated glomerular filtration rate; TERT, telomerase reverse transcriptase.

CME can be divided into five sequential steps: initiation, progression, growth, fission, and uncoating (68, 69). A thorough discussion of the importance of phosphoinositides (PIs) is beyond the scope of this review. but these phospholipids, while representing <10% of membrane phospholipids, have an ever increasingly understood role in endocytosis (78). Note that the location of some key PIs is indicated in FIGURE 5. Their roles in actin regulation, cell signaling, budding, and fusion of transport carriers have established the spatiotemporal control of specific PIs, and their conversion creates membrane nanodomains necessary for multiple cell biological pathways such as receptor sorting and budding of an endocytic vesicle. Mutation in the inositol polyphosphate 5-phosphatase OCRL is known to cause disruption in endosomal trafficking and alterations in primary cilia assembly as observed in Dent disease and Lowe syndrome (79, 80). The phosphatase INpp5B, a paralog gene of OCRL, may also have a role given its ability to partially compensate for loss of OCRL1 in knockout mice (80). A recent study used a Rab11 biosensor to show that PI3KC2α controls Rab11 activity in peripheral endosomes with active Rab11 recruiting the PI(3)P phosphatase (MTM1) leading to triggering the fission of endosomal tubules or vesicles with recycling endosomes (81). Similar PI conversions occur to regulate other budding and fission events during CME. In addition, a role for PIs in clathrin-independent and dynamin-independent endocytic pathways has been shown (82). Although the PIs have their important roles throughout CME, the initial clustering of receptors and formation of clathrin-coated pits (CCPs) is dependent upon short cytoplasmic motifs, i.e., YXXΦ, in the receptors that bind directly to adaptor proteins such as AP2 that contain phosphotyrosine binding domain (PTB)/phosphotyrosine interaction domain (PID) domains (69). The constitutively internalized receptors TfnRs and LDLRs are internalized independent of ligand binding. The AP2 complex binds to phosphatidylinositol 4,5-bisphosphate PI(4,5)P_2_ and PTB/PID motifs triggering clathrin assembly and recruiting additional endocytic accessory proteins to complete initiation and stabilization. Structural studies have shown the AP2 complex undergoes a conformational change upon membrane interaction that opens up the structure, exposing the clathrin binding site leading to the stabilization of forming CCPs. This open conformation is enhanced by F-BAR (Bin/Amphiphysin/Rvs) domain-containing FCH01/2 proteins and additional adaptor proteins such as PICALM that function as adaptor proteins for soluble *N*-ethylmaleimide-sensitive factor attachment protein receptors (SNAREs) (69). This is important for targeting and fusion. Many of these interactions, although important, are of low affinity and also have regulation by phosphorylation and glycosylation, making deciphering specific ligand and receptor pathways challenging (83, 84).

The growth and maturation of the CCP involves cargo loading, increased curvature, and PIP conversion. A key protein in maturation and fission of CCP is Dynamin, whose *Drosophila* homolog, shibre, was found to have substantial blocks in synaptic endocytosis that by EM suggested membrane fission blocks (69, 85). Subsequent studies placed dynamin in CCPs at all stages, although its precise role in early stages is unclear. Dynamin is a GTPase with five domains, and its complex regulation involves phosphorylation of its proline/arginine-rich domain (PRD), which modulates its binding to up to 13 different SH3 domains shown to have unique effects on its assembly and activity. These SH3-containing proteins also contain other domains that interact with other coat constituents and actin regulators that together provide multiple PTM and allosteric opportunities for unique regulatory steps to optimize a specific cells function(s). It is also becoming evident that dynamin isoforms are regulated differently, and studies to explore these differences in the PTCs are needed (86). PIK3C2A interacts with clathrin and alters the phospholipid environment, enabling recruitment of SNX9, which interacts with dynamin and actin-branching proteins to facilitate constriction (82). Fission serves to release or form the early endosomes that are located adjacent to the apical or basolateral membranes. This process also involves release of the clathrin coat facilitated by the uncoating chaperone HSPA8 (82).

Newly formed endosomes can be sorted by four primary pathways: *1*) recycling to apical plasma membrane via a Rab4 dependent pathway, *2*) recycling to apical plasma membrane after transit through Rab5 and 7 endosomes and a sorting/recycling compartment via a Rab11 pathway, *3*) delivery to lysosomes for degradation via Rab5 and 7 endosomes, and *4*) transcytosis following trafficking to the sorting/recycling compartment, also via a Rab11 pathway (71, 82, 87–89). A common theme in eukaryotic vesicular transport is the participation and regulation by one or multiple members of the large Rab family, containing nearly 70 members (90). These small GTPases function by cycling between the active GTP-bound membrane form and the inactive GDP-bound cytosolic form. The activation/inactivation of the Rabs is tightly regulated by three classes of proteins: *1*) guanine nucleotide exchange factors (GEFs), *2*) GTPase activating proteins (GAPs), and *3*) GDP-dissociation inhibitors (GDIs) (91). The Rabs, in essence, function as on/off switches serving to dictate where specific vesicles are targeted. There is a host of additional effector molecules that provide additional specificity via their different domains. Although there is still much to decipher, some common roles for Rabs and some specific targeting mechanisms have been defined (90–92).

Characterization of the early endosomes (EEs) in different tissues and cell types is under active investigation, and some common attributes have been identified (82, 87). First, EEs often have a high concentration of phosphatidylinositol 3-phosphate (PI3P) and Rab5 along with Rab5 effectors and PI3P interacting proteins (90). Rab4 is also found on early and recycling endosomes. Rab5 can recruit phosphatidylinositol (3,4,5)-trisphosphate (PIP3) kinase, thereby increasing PI3P, which in turn recruits FYVE domain-containing proteins such as faciogenital dysplasia (Fgd) family members (93). Fgd proteins are unique in their structure, containing multiple domains including pleckstrin homology (PH), FYVE, and a Dbl homology (DH) domain. PH and DH function together to activate Rho proteins by catalyzing the exchange of GDP for GTP nucleotides. Other FYVE binding proteins found on EE include EEA1 (a Rab5 effector), rabenosyn-5 (a Rab4/Rab5 effector), and other Rab members. These associations make the EE very dynamic in movement and promote fusion with newly formed endocytic vesicles. Early endosomes lead to the formation of the sorting endosome (SE), which in proximal tubule cells consists of both an apical SE (ASE) and a basal SE (BSE) (87). Sorting endosome compartments are located close to the apical or basolateral membranes and represent the first sorting station for determining whether cargo will be recycled, delivered to a common recycling endosome (CRE) that receives cargo from both apical and basal lateral membranes, or targeted to the degradation lysosome pathway. Current evidence supports that both clathrin and nonclathrin endocytic mechanisms deliver their cargo to the SE compartments. Sorting involves membrane heterogeneity, which in turn determines which Rabs and associated effectors are recruited. Rabs 4, 5, 7, and 11 have been found on SE compartments with cargo destined for late endosomes and lysosomes associated with Rab7, whereas those destined for the CRE utilize Rab11. Studies using polarized MDCK cells stably expressing mini-Megalin (a truncated form of the protein containing the full cytoplasmic tail) have shown that internalized megalin is first located in ASE and is then delivered to the CRE, which also receives basolateral endosomes such as the transferrin receptor TfR (94). Recycling of megalin to the apical membrane from the CRE requires Rab11 and was found to be reduced by ∼40% when cells were treated with nocodazole, implying that it is in part microtubule dependent. Note that MDCK cells were found not to contain megalin or cubilin transcripts, emphasizing the importance of cell line characterization and interpretation of results (73).

Soluble *N*-ethylmaleimide-sensitive factor attachment protein receptors (SNAREs) also provide specificity to the endosomes. Thirty-eight SNARES have been identified, with 30 being located to endosomes and/or phagosomes (95). Although beyond the scope of this review, it is important to note that a specific set of SNAREs on each membrane form a SNARE assembly driving membrane fusion. The membranes destined for degradation remain in Rab5 sorting endosomes that can undergo a switch to Rab7 positive endosomes. This switch involves the recruitment of Rab effectors such as MON1A, VPS34, and RILP (96). These steps will ultimately dictate whether the membranes and associated cargoes are trafficked to lysosomes for degradation or enter the retromer/retriever pathway destined for the *trans*-Golgi network (TGN).

Endocytic cargo that will be recycled can be internalized by both clathrin-dependent and clathrin-independent pathways and in either case rapidly appear in Rab11 compartments. The makeup of the membrane is believed to have a major role with phosphatidylserine (PS) being enriched in the Rab11 recycling pathway (82). Studies in multiple systems document that Rab11 compartments can receive endocytic material from both apical and basolateral membranes in the CRE (97). Cargo exiting to the plasma membrane from these Rab 11 sorting endosomes requires binding of Rab11-family interacting proteins (Rab11-FIPs) to PS membranes and recruitment of dynein, SNAREs, Eps15 homology domains (EHDs), VPS, and SNX proteins. Interestingly, PS membrane domains can induce endocytosis without clathrin-dependent or clathrin-independent regulators (98). Flotillins are also thought to maintain the PS microdomains and appear to accumulate in Rab11 endosomes. One Rab11 GEF that is enriched in the S1 tubule, Rab3IL1, may link Rab11 with trafficking processes of Rabs 3 and 8 (99). It has also been shown that plasma membrane recycling is necessary for surface homeostasis. In polarized cells like the PT recycling of macropinocytosed membranes may be a major pathway (100).

### 3.2. Clathrin-Independent Endocytosis

Although CME is by far the best-studied method of endocytosis, less-defined mechanisms of clathrin-independent endocytosis (CIE) exist in many cells (82). Two general types of CIE have been defined, dynamin dependent and dynamin independent, with initiation of endocytosis in all cases being dependent on the local microenvironment including membrane heterogeneity at the site of internalization (82, 101). Evaluation of each type of CIE is still in the early stages, but three types of dynamin-dependent CIE have been defined: *1*) activity-dependent bulk endocytosis (ADBE), *2*) ultrafast endocytosis (UFE), and *3*) fast endophilin-mediated endocytosis (FEME). ADBE occurs in neurons along with UFE, which mediates the recycling of synaptic vesicle components. FEME is a nonconstitutive endophilin (member of the BAR domain superfamily)-regulated pathway. Endophilin performs three critical functions for FEME: *1*) BAR domain promotes membrane curvature, *2*) cargo interaction via its SH3 domain, and *3*) recruits actin and dynamin, leading to membrane scission. Receptors that utilize FEME include epidermal growth factor receptor (EGFR) and IGF-1 receptor (IGFR), and some evidence exists for the scavenger receptor A and CD36 using this pathway. It appears that some receptors can utilize multiple endocytic pathways. Understanding what regulates the specific pathway chosen requires further studies.

Three dynamin-independent CIE pathways that have been defined include *1*) clathrin-independent carrier (CLIC)/GPI-anchored protein (GPI-AP)-enriched early endosomal compartments (GEECs), *2*) massive endocytosis (MEND), and *3*) macropinocytosis (82, 101, 102). CLIC-GEEC endocytosis is a constitutive process that appears to be triggered by the clustering of GPI-APs or glycosylated proteins and lipids cross linked by galectins (103). It does not require dynamin, but instead membrane curvature appears to be induced by a dynamic interplay between cholesterol and phosphatidylserine. Cholesterol is also required for CDC42 activation leading to actin polymerization and recruitment of BAR-domain proteins and the actin nucleation complex. This triggers the characteristic membrane tubulation observed in this mechanism. In contrast, MEND involves Ca^2+^ and phosphatidylinositol 3-kinase (PI3 kinase) signaling and is dependent upon membrane phase separations (101, 104). A role for clathrin, dynamin, or actin has not been found; however, protein palmitoylation does appear to be important and may involve CoA and acyl CoA release from mitochondria (105). Interestingly, a potential master regulator of CoA biosynthesis, PANK2, was found enriched in the S1 tubule segments (see TABLE 2). The participation of membrane phase separations in multiple systems is increasing, and while still not widely accepted they appear to participate in membrane budding and fission at both the plasma membrane and internal membrane components (104). The involvement of these CIE mechanisms in PT uptake deserves further investigation. Interestingly, an early immune-EM study showed that megalin was restricted to microvilli and coated pits and not present in the larger vacuoles and apical tubule structures (106). It has been suggested that the physical formation of larger vesicles is more likely to occur without clathrin (104); thus might these unique structures present in the PTs (apical tubules and vacuoles) form in part from the contribution of a CIE mechanism.

## 4. ALBUMIN

**FIGURE 6.**
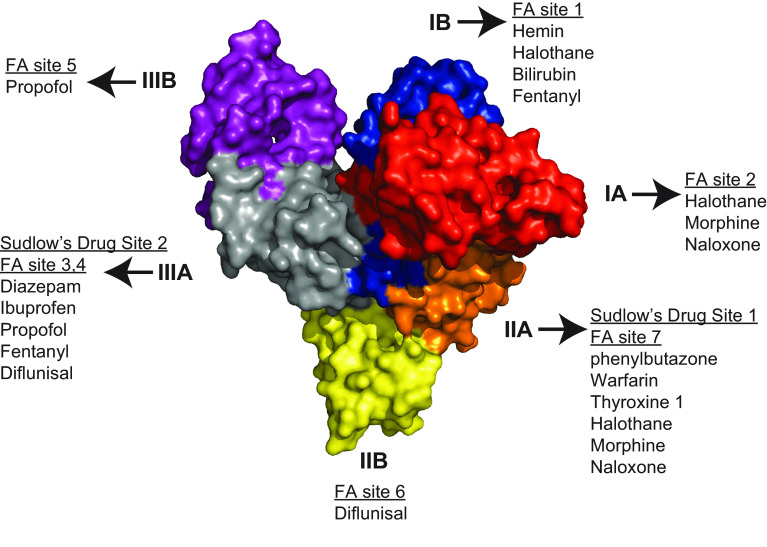
Albumin’s structure, domains, and binding sites. Albumin domains are color coded, and fatty acid (FA) binding sites and physiologically relevant known drug sites are highlighted in Sudlow sites I (DIIA) and II (DIIIA) and other subdomains of albumin. The domains are color coded: red, IA; blue, IB; light brown, IIA; yellow, IIB; gray, IIIA; purple, IIIB. Data from Ref. 107.

Albumin is a protein composed of three homologous domains, has a molecular mass of ∼66 kDa, and interacts with a wide variety of ligands (FIGURE 6) (107–109). It is the most abundant serum protein and has many important physiological functions including colloid osmotic pressure, antioxidant, pH buffering capacity, and transport of endogenous and exogenous compounds including free fatty acids (FA), hematin, bile acids, copper, and a number of therapeutic agents. It is produced exclusively in the liver, although minute quantities of mRNA lacking a normal-length polyadenylate tail have been found in the kidney (110) and may be activated after acute kidney injury (AKI) (111). Albumin carries 99% of long-chain fatty acids (LCFA) in serum with a usual molar ratio of two LCFA per albumin molecule. This can increase to 4:1 with exercise, and the maximum carrying capacity is 6:1. Domain 3 of albumin has the highest affinity for LCFA and binds two per molecule. Dissociation of LCFA occurs rapidly in an acid environment with a p*K*_a_ of 4.8 and accounts for delivery of LCFA to numerous tissues (112). The β-oxidation of the released fatty acids in the mitochondria is also used to generate ATP (113). Interactions and noncatalytic reactions within the serum alter albumin’s physical properties, and these interactions are known to increase in certain disease states. Glycosylation of albumin is increased with increasing serum glucose concentration and increases the susceptibility to diabetic complications including nephropathy (114–116). Albumin carbamylation occurs from a noncatalyzed reaction with urea and is proportional to serum urea concentrations. Albumin is metabolized by a wide variety of tissues, with most of the metabolism occurring in muscle and skin in rats. The kidney is only responsible for ∼10% of total albumin metabolism (117, 118). Once internalized into cells, such as skeletal muscle cells, modified albumins are degraded whereas physiological albumin is released intact (112). The clinical importance of these modifications is discussed below.

Albumin degradation can occur in multiple sites after glomerular filtration. After glomerular filtration, albumin can undergo hydrolysis by apical membrane proteases within the lumen of the PT or be reabsorbed. After PT internalization, albumin can undergo transcytosis or lysosomal digestion with either reclamation of the amino acids or exocytosis of peptides back into the glomerular filtrate (119–121). Initially, albumin reabsorbed by PTs was believed to be metabolized in the lysosome to amino acids for reutilization (32). Isolated perfused rat kidney studies (122), HK-2 cultured cell studies (119), and in vivo rat models have demonstrated that albumin can be rapidly hydrolyzed into small peptides and amino acids released into the tubular lumen (123). Proteases at the apical membrane can also hydrolyze filtered albumin and peptides into fragments (124, 125). However, a note of caution is necessary. Comparing in vivo endocytosis with cultured cells may not be appropriate. Proximal tubule cells in vivo undergo apical endocytosis at a much more rapid rate than cultured PT cells (25, 126, 127). Also, PT apical endocytosis is many times greater than basolateral endocytosis in vivo, but the two are equivalent in cultured cells (25, 127, 128). The metabolic rate of cultured cells is also much less than that of PT cells in vivo. Finally, transcriptomic profiling of multiple PT cell lines showed that none matched the transcriptome of native PTs, the highest match from opossum kidney (OK) cells at only 45% (73).

Albumin serves many beneficial functions [maintaining plasma osmotic pressure, transporting vitamins, fatty acids (FA), bile, sequestering toxins, antioxidant, etc.] under physiological conditions, but in disease states albumin can become modified and/or its normal metabolism can be altered, contributing to dysfunction. Albumin is known to cause PT stress, injury, interstitial cell infiltration, and fibrosis through a large number of cellular toxicity pathways (129). These pathways include complement activation, chemokine expression, NF-κB-dependent and -independent pathways (130, 131), oxidative stress with superoxide generation via NAPDH oxidase activation with subsequent generation of H_2_O_2_ and multiple intracellular pathways (132, 133), type II TGF-β upregulation (134), endoplasmic reticulum (ER) stress with caspase 12 activation and apoptotic cell death (135), dysfunctional autophagy (136), inflammasome activation (137), delivery of nonesterified fatty acids by excess filtered albumin resulting in apoptosis (138), and Klotho downregulation leading to increased FGF 23 expression (139). Zoja et al. (140) have reviewed this topic and offer an integrated approach, yet what remains to be determined is exactly what is leading to the toxicity of urinary proteins and in particular albumin. As discussed below, modifications to albumin and the cargo bound to albumin may play important and poorly understood roles in initiating these injury cascades.

## 5. GLOMERULAR FILTRATION OF ALBUMIN

**FIGURE 7.**
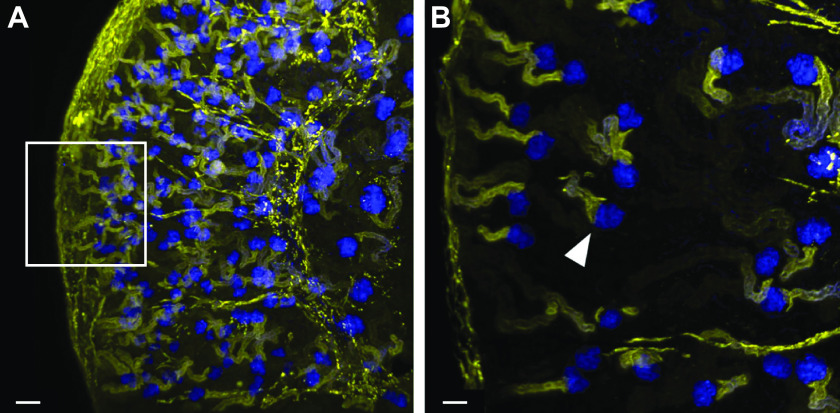
Light sheet fluorescent microscopy and 3-dimensional image reconstruction image of a tissue-cleared mouse kidney at low (*A*; bar, 100 µm) and high (*B*; bar, 50 µm) magnification. Male C57BL/6 mice were injected with DyLight-649-tomato lectin (blue) and Alexa Fluor 555-albumin (yellow). Note that lectin labels glomeruli and filtered albumin is taken up by proximal tubules. Arrowhead in *B* represents the glomerulotubular junction. Figure from Ref. 144, with permission from *Kidney360*.

Although it is universally accepted that albumin is filtered, the extent of albumin filtration across the glomerulus remains controversial. Although this is not a primary objective of this review, it is important to discuss the controversy. Numerous reviews highlighting both sides of the controversy are available to the reader (2, 4, 141, 142). Briefly, previous micropuncture studies in rats and mice revealed a very low glomerular sieving coefficient of albumin (GSCa, the ratio of Bowman’s space albumin to plasma albumin), in the range of 0.005 ± 0.005, whereas our multiphoton microscopy studies of fluorescent albumin quantified a GSCa in Munich Wistar Frömter rats of 0.012 ± 0.003 (4). In Munich Wistar Simonsen rats we quantified a GSCa of 0.030 ± 0.005 (143). Other laboratories utilizing fluorescent albumin in multiphoton microscopy intravital studies and light sheet fluorescent microscopy coupled with automated three-dimensional (3-D) histomorphometric analysis in tissue-cleared kidney, also observed a high level of filtered fluorescent albumin (FIGURE 7) (20, 144). Since the field was relatively noncontroversial until these multiphoton data were published, the discussion presented here centers around responses to challenges of the multiphoton data, using published data to support the accuracy.

The observation of increased glomerular permeability of serum albumin, and a greater role of proximal tubules in its reclamation, sorting, and transcytosis back to the bloodstream, by two-photon microscopy is an example of how a new technology generating a paradigm shift is often met with skepticism (145). After two initial publications by Russo et al. (38, 143) delineating an increased glomerular sieving coefficient for albumin, publications by different independent investigators questioning the observations based on their own follow-up studies were published (146, 147). Differences and difficulties explaining their inability to repeat the Russo et al. observations can be grouped into two areas. The first area involves the materials used to quantify GSCa and how they were administered during the intravital imaging studies (4). The second area encompasses the technical aspects of the two-photon systems used to collect the data and how certain parameters are essential to allow detection of low-intensity fluorescent signals within Bowman’s space (2, 148). Ultimately, regardless of the GSCa values reported, the accumulation of fluorescent albumin in renal proximal tubules indicates filtration into Bowman’s space with subsequent binding at the apical brush border, and internalization occurs in all nephrons within the kidney (FIGURE 7).

The importance of using labeled albumin and not a widely dispersed dextran with an average molecular weight similar to albumin was shown (4). Studies by Pedi-Peterdi et al. (147) used a broadly dispersed 70-kDa rhodamine dextran, and not fluorescent albumin, in an attempt to quantify the GSC of albumin. This commercially available dextran produces time-dependent GSC values, as low-molecular-weight molecules are filtered rapidly and dextran molecules larger than albumin are filtered at a slower rate. Therefore, the GSC determined depends on when the measurements are taken. These differences can span several orders of magnitude (4). A subsequent study suggested that the high GSCa values were due to an underestimation of the true plasma fluorescence of labeled albumin (149). In this study the measured GSC of FITC-inulin, which is known to have a GSC of 1.0 and be freely filtered, was evaluated. Administration of the inulin was accomplished with a single bolus intravenous injection, and measurements were taken within the first 40 s. This produced a GSCi value of 2.12 ± 0.16 where the Bowman’s space fluorescence was much higher than the plasma fluorescence. This occurs because the bolus load is not distributed evenly within the plasma when it is mixing after injection. This produces rapidly falling plasma values while Bowman’s space values and therefore GSC values lag and remain artificially high because of the rapidly decreasing plasma fluorescence levels. The distribution of fluorescent inulin, or for that matter any molecule, must come to a stable plasma equilibrium value before the GSC is determined. For inulin this occurs after minutes of continuous infusion, not seconds (143, 150).

As with any digital/electronic system, signal quantification is greatly affected by the sensitivity settings used for the system detector. With microscopy, to ensure maximum sensitivity the detector’s offset should be set to where only a few random pixels are producing values of zero at baseline. An intuitively easier option would be to set all the background values in the areas of interest to zero, which would negate the need to collect background values. However, this reduces the detector’s sensitivity, minimizes low-level fluorescent detection, and results in a lower GSCa (151). Interestingly, the use and result of too high offset values can be observed from one study examining fluorescent albumin filtration (152). In this publication a background image is shown in one of their figures indicating that the background settings used for Bowman’s space were all zero. This reduced their sensitivity and therefore the ability to detect fluorescent albumin in Bowman’s space, resulting in a low GSCa. In another study, the offset was adjusted in the detectors before and then readjusted after infusion of fluorescent albumin (149). This approach invalidates any data, as the background images will no longer be subtracted out to correct for preexisting values. Another technical aspect called into question regarding the high GSCa values is the use of an 8-bit detector system (256 gray levels from black to white) instead of the then-emerging 12-bit detectors (4,095 gray levels from black to white) (148). The publication by Sandoval et al. (148) addresses the technical aspects of photon detection and bit depth. The same laboratory has used four different microscope systems over time, all with increasingly superior components, and all have produced the same GSC values for albumin (3, 4, 6, 38, 143, 151, 153–155).

Finally, an additional study (150) suggested that out-of-focus fluorescence emanating from both above and below the image focal plane was responsible for producing the high Bowman’s space fluorescence values, and that this could be avoided by using internal (descanned) photodetectors. However, early work from investigators studying two-photon excitation (156, 157) described the physics of two-photon excitation and how the optical components used in standard single-photon confocal microscopy decrease light collection efficiency. Briefly, multiphoton excitation of fluorescent molecules occurs when two or more low-energy (far red) photons stimulate a fluorophore, causing a jump to a higher energy state and emission of a higher-energy photon (such as a green FITC photon). Longer-wavelength excitation photons are delivered in short, pulsed packets and only at the focal plane of the objective is the photon density high enough that excitation probability occurs. Above and below the focal plane photons pass through the sample, causing no fluorophore excitation. Using less efficient internal (descanned) detectors (which have a longer light path and pinhole) reduces photon collection efficiency compared with external (nondescanned) detectors. Nondescanned detectors have a shorter light path (they can be placed closer to the sample) and use no pinhole, factors that increase the collection efficiency of emitted light from the biological sample. Images taken during all of our studies for GSCa determinations, or when studying other parameters, are typically taken at an average depth of 5–30 µm when extracting numerical data from the images and deeper for qualitative/scoring analysis. To restate, fluorophore excitation occurring only at the objective focal plane drives this technology.

These two parameters, the use of descanned instead of nondescanned detectors with better light collection capabilities and having offset settings adjusted too high (decreasing sensitivity) on photodetectors, can produce an artificially low albumin GSC value. Close inspection of the methods section or figures in these manuscripts will reveal the points made in this brief section. Despite this, some investigators have persisted in using suboptimal settings with lower sensitivity (158).

## 6. ALBUMIN ENDOCYTOSIS BY PROXIMAL TUBULE: MECHANISM

Albumin arrives at the PT carrying bound ligands and modified amino acids depending upon interactions and modifications occurring in the serum. Thus, filtered albumin presents to the PT a unique sampling of the body’s physiological state. How the PT responds to normal and to these modified albumins will be dictated first by whether the albumin is internalized, second by what receptor binds to the respective albumin, third by the subsequent receptor signaling induced, and fourth by its subsequent trafficking. Each of these steps requires unique interactions dictated in part by albumin state.

**FIGURE 8.**
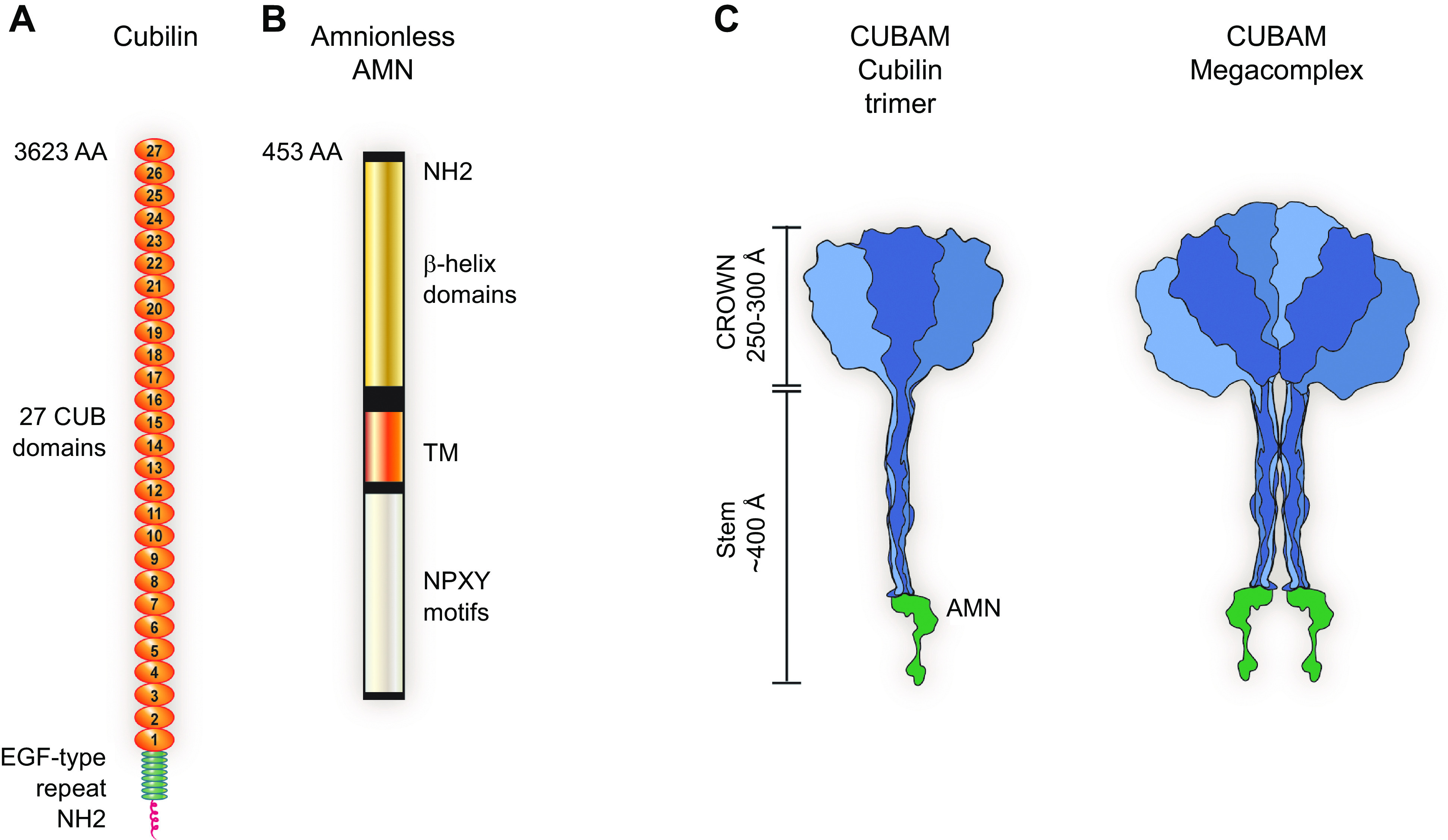
Cubilin and Amnionless domains and structural complex. *A*: Cubilin is a peripheral membrane protein containing an NH_2_-terminal stretch of 110 amino acids (AA), 8 epidermal growth factor (EGF)-type repeats, and 27 CUB domains (162). *B*: Amnionless is a transmembrane protein with a cytoplasmic domain of 75 amino acids containing 2 putative NPXY motifs followed by a transmembrane region (TM) and a cysteine-rich region that links to the NH_2_-terminal part of AMN that form 2 β-helix structures with hydrophobic cores. *C*: single-stem form of CUBAM with approximate dimensions of stem and crown regions followed by a representation of the double-stem form of CUBAM. Data from Ref. 163.

The mechanism of PT albumin uptake has been investigated in both in vivo and in vitro systems, and the data support that PT albumin endocytosis occurs via direct interactions with cubilin and indirect interactions with megalin (71). Cubilin (CUBN) was first defined as the receptor for vitamin B_12_, a critical function given that biosynthesis of B_12_ is restricted to prokaryotes (159, 160). These studies led to the purification of cubilin and identification of its domains (FIGURE 8*A*) and its calcium-dependent interaction with megalin (161).

Subsequently, it was found to have a novel interaction with the type 1 transmembrane protein amnionless (AMN) (FIGURE 8*B*) to form the Cubam complex (FIGURE 8*C*) (164). Increased understanding of the Cubam complex has come from genetic analyses of Imerslund–Grasbeck syndrome (IGS) and juvenile megaloblastic anemia (MGA1), which are caused by mutations in either AMN or CUBN (165–168). These studies established that the AMN and CUBN interaction was essential for cell surface targeting of the Cubam complex and identified four cubilin *N*-glycosylation sites necessary for surface expression (169, 170). The biosynthetic pathway of cubilin requires the correct folding of cubilin in the endoplasmic reticulum (ER), which is dependent upon proper *N*-glycosylation and interaction with the quality control check lectin chaperones calnexin/calreticulin and glycosyltransferase Uggt1 (171). In addition, 39 patients with biallelic pathogenic variants in the CUBN gene were found to be associated with chronic isolated proteinuria (165). Interestingly, four COOH-terminal CUBN variants were associated with albuminuria.

Cubilin contains a coiled-coil NH_2_-terminal region and 8 epidermal growth factor-like (EGF-like) domains followed by 27 CUB (complement C1r/C1s, Uegf, Bmp1) domains (159, 172, 173). Protein expression and analyses of specific cubilin domains showed CUB5–8 to contain the intrinsic factor (IF)-B12 and albumin binding sites. When IF-B12 is bound to CUB5–8 albumin binding is reduced, whereas IF-B12 binding was not altered by albumin binding (173). These series of studies also identified Alpha-2-macroglobulin receptor associated protein (RAP) binding domain (CUB13 and 14) and found three regions (NH_2_ terminus + EGF + CUB1 and 2, CUB12–17 domain, and CUB22–27 domain) of cubilin that bound to megalin in a Ca^2+^-dependent manner (161, 172). Additional studies are needed to define the interaction between cubilin and megalin and how this is regulated. This is especially important given the multitude of ligands these two large proteins bind. In addition, cubilin exists as a trimer at the cell surface because of self-association of an α-helical coiled-coil structure near its NH_2_ terminus forming a single intertwined β-helix domain that docks to a three-faced β-helix domain in AMN (FIGURE 8*C*) (163, 174). This cubilin-AMN megadalton receptor could have multiple binding sites for albumin, and its other ligands, although how this structure affects binding affinity and ligand release is unknown.

Evidence that both cubilin and AMN can undergo proper membrane localization and albumin endocytosis independent of megalin was first shown in CHO cells (175) and more recently in the HCT116 colorectal cell line (176). This study also identified the nuclear valosin-containing protein-like 2 (NVL2) as an AMN interacting protein facilitating CUBAM-mediated albumin endocytosis (176). Two adaptor proteins, Disabled-2 (Dab2) and Low-density lipoprotein receptor adapter protein 1 (LDLRAP1), bind to NPXY motifs on AMN and mediate uptake of CUBAM ligands (71, 175). Although simpler cell systems, including cell culture systems, can help establish minimal associations necessary for specific events, i.e., albumin endocytosis, deciphering whether these same associations are sufficient in vivo is a more challenging question. The proximal tubule cell, with its highly organized and specialized apical domains, may restrict or enhance interactions based on location, concentration, and a multitude of regulatory processes not fully represented in some of the in vitro cell culture models (73). For instance, both glycated and carbamylated albumin have shown reduced to no binding to cubilin’s albumin binding domain, yet PT uptake of these modified albumins is unaltered when quantified by rat intravital imaging (6, 155). Understanding how this is occurring in vivo is important. For example, whether cubilin has additional albumin binding sites or another uptake mechanism is involved is an important question for future investigation. A discussion of specific gene manipulations and human mutations impacting PT function and albumin handling is presented below. These investigations can reveal novel insight into cellular processes but can also provide misinformation if overinterpreted. One caveat is that total knockouts and even gene truncation mutations can have off-target consequences, induce compensatory mechanisms, and even trigger expression of related genes (177–180). Ultimately, the analysis of complementary approaches including perturbation, visualization, substitution, characterization, reconstitution, and simulation will provide a more complete and accurate representation of the role of individual molecules in these complex and dynamic cellular systems (181).

Megalin is the largest member of the low-density lipoprotein receptor family (LDLR), >500 kDa, and was first identified as the pathogenic antigen in Heymann nephritis (182). Immunolocalization studies show localization in PTs primarily at the apical surface and in apical endocytic compartments (21, 35, 71, 94). More recent RNA sequencing (RNA-seq) and proteomic analyses of isolated rat tubule segments suggest that S2 tubules contain the highest concentration of megalin, with slightly lower concentration in S1 and S3 segments (74, 75). Additional localization studies using epitope-specific antibodies and analysis in different mammalian kidney tubules would define a more complete picture of this receptor’s localization. However, there is no question of the importance of megalin in capturing and via receptor-mediated endocytosis recovering many filtered molecules including enzymes, low-molecular-weight proteins, Ig light chains, vitamins, and hormones (35, 71). Our focus is on albumin uptake, and the most-cited data supporting a direct interaction of megalin and albumin consist of a result from passing albumin over a megalin column with only 7% and 13% of albumin being bound (183). Consequently, the consensus thinking is that megalin internalizes albumin via its direct interaction with cubilin, which does directly bind albumin. Whether megalin is able to bind modified albumins such as glycated or carbamylated albumin has not been determined.

**FIGURE 9.**
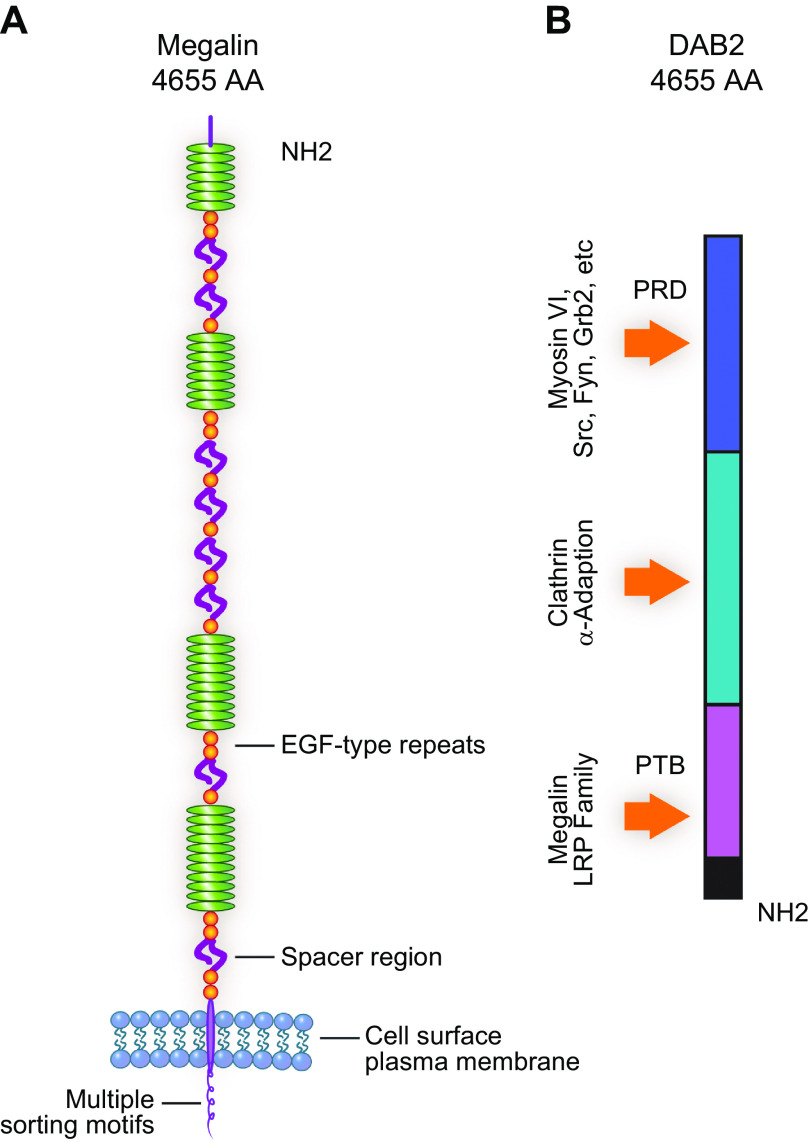
Megalin and DAB2 domains. *A*: Megalin is a large, 4,655-amino acid (AA), transmembrane protein with an extracellular domain that consists of 4 clusters of complement-type repeats, separated by 8 spacer regions containing YWTD motifs and 17 epidermal growth factor (EGF)-type repeats (162). Its cytoplasmic tail contains multiple sorting motifs including PDZ, PKC, SH3, and NPXY (188). *B*: DAB2 is composed of 3 principal domains. The NH_2_-terminal PTB domain binds to NPXY motifs, the middle domain interacts with clathrin and alpha-adaptin, and the COOH-terminal portion is a proline-rich domain (PRD) that can bind SH3-containing proteins such as Grb2, Fyn, and Src (189).

Megalin is a type I transmembrane protein containing four ligand binding domains each made up of nine low-density lipoprotein receptor (LDLR) type A repeats separated by EGF-type repeats and eight β-propeller spacers containing YWTD motifs (FIGURE 9*A*) (162). The YWTD domain undergoes a structural change in acidified compartments, thus mediating ligand dissociation (184). Megalin’s biosynthetic trafficking and folding is dependent upon the molecular chaperone Alpha-2-macroglobulin receptor-associated protein (LRPAP1 or RAP), which resides in the ER and binds to megalin’s ligand binding domains, thus preventing premature ligand binding that would result in megalin degradation (185, 186). The chaperone MESD is also required for proper folding of the six-bladed β-propeller domain contained in the EGF domain regions of megalin (187). The megalin-RAP complex is trafficked to the *cis*-Golgi compartment, where RAP is released because of a lower pH and recycled back to the ER. Megalin also contains the longest cytoplasmic tail of LDL receptor members consisting of the following motifs or domains: LL, 2-SH3, 2 NPXY, 1 NPXY-like, PKC, and PDZ (188). Studies using polarized MDCK cells stably expressing mini-megalin have shown an important role for the NPXY-like site and interaction with both clathrin and adaptor protein-1 in apical delivery of megalin (188). In addition, phosphorylation of a PPPSP motif in the cytoplasmic tail may also regulate recycling and surface expression (190, 191).

Although the megalin cytoplasmic tail is quite divergent from other members of the LDL receptor family, it does contain two NPXY motifs that mediate endocytosis. The NPXY motif binds to the phosphotyrosine-binding domain (PTB/PID) on Disabled-2 (Dab2) and LDLRAP1 located at their COOH terminus (192, 193). An important role for LDLRAP1 in megalin endocytosis was reported by the Farquhar laboratory, and multiple roles for endocytic adaptor proteins in different human diseases are becoming apparent (194, 195).

Dab2 is an endocytic adaptor protein with similarity to the Disabled protein in *Drosophila* involved in embryonic neural development (196). Because of its multiple protein binding motifs (FIGURE 9*B*), it has been shown to participate in the formation of multiprotein complexes important for clathrin-mediated endocytosis (CME) (197). Dab2 also contains clathrin binding, DPF, and NPF motifs that bind endocytic vesicle components clathrin, EPS-15, and alpha-adaptin with the COOH terminus having a Myo6 binding site (189). Mice lacking Myo6 were shown to have impaired receptor and fluid-phase endocytosis (198). These multiple sites allow Dab2 to link megalin and its associated ligands to the clathrin coat and myosin motor facilitating their endocytosis and trafficking (189). Dab2 also has two isoforms, p96 and p67, both of which are present in the kidney (193). Additional roles for Dab2 in multiple diseases and other cellular functions including immune function, cellular homeostasis, and inflammatory processes have been found, suggesting that it has additional roles in the PT (197).

## 7. PHYSIOLOGY OF ALBUMIN REABSORPTION BY PROXIMAL TUBULE CELLS

The dogma regarding albumin filtration and reabsorption states that a small amount of albumin, ∼3–5 g/day, is filtered across the glomerulus and then reabsorbed and catabolized by PT, primarily the S1 segment, where CME is greatest. This dogma has been challenged by Comper with a series of biochemical approaches to evaluate albumin filtration and PT reabsorption (see for reviews Refs. 199–201).

Albumin was shown to be transported across PTs in proximal tubule microperfusion studies (32, 125). Controversy existed around this finding until Russo et al. (143) showed rapid apical uptake, intracellular trafficking, and basolateral release of albumin with two-photon and electron microscopic techniques. This was validated by an elegant molecular approach using a transgenic mouse with podocyte-specific expression of an inducible tagged murine albumin secreted into the filtrate. Induction of the tagged albumin, either a neutral or negatively charged albumin, resulted in serum accumulation of the albumin and proof of transcytosis. A transgenic deletion of FcRn abolished transcytosis and serum accumulation of the tagged albumin, indicating the critical role of FcRn in albumin transcytosis (5). Russo et al. followed up on their transcytosis study with a study in streptozotocin-induced diabetic rats. This study indicated that the increase in albuminuria was due to reduced PT albumin reabsorption and not a change in the glomerular sieving coefficient, a measure of glomerular permeability (38). A previous study in proteinuric diabetic rats also showed reduced tubular reabsorption of albumin associated with decreased height of apical membranes (202). A potential mechanism underlying reduced albumin endocytosis by PTCs in patients with diabetes was identified in cultured LLCPK cells as a reduction in megalin function induced by high glucose (203). These observations were further supported in an inducible megalin knockout mouse model where the efficiency of albumin reabsorption was reduced in diabetic compared with nondiabetic mice (204). This same study showed the that inducible knockout of megalin was seen in 92% of PT and resulted in urinary albumin increasing from <10 µg/24 h to >250 µg/24 h, showing the importance of PT albumin reabsorption in normal physiological state and its dependence on megalin. In the most recent study showing a high glomerular filtration and reabsorption of albumin in physiological states, Ostergaard et al. used tissue clearing techniques to qualitatively and quantitatively evaluate albumin filtration and uptake by PT. C57BL/6J mice were injected with DyLight-649-conjugated tomato lectin to label glomeruli and Alexa Fluor 555-conjugated BSA mice to show albumin glomerular filtration. Two to three minutes after the injection mice were perfused fixed (144). Kidney tissue was cleared and imaged on a light sheet fluorescent microscope obtaining full-thickness images. Large quantities of albumin were seen in virtually all S1 and S2 PT segments (see FIGURE 7). This approach provides a global view of the extent and rapid nature of albumin filtration and highlights the quantity of albumin filtered across the glomerulus under physiological conditions.

The reabsorption of albumin by PT occurs by both high-affinity, low-capacity and low-affinity, high-capacity transport systems (32). The megalin-cubilin receptor complex is well studied, and a myriad of reviews have been written to describe its function and role in protein absorption and metabolism (71, 142, 205, 206). The dissociation coefficient (*K*_d_) of albumin to cubilin is very low and is estimated at 0.63 µM at pH 7.0 (207), resulting in a high-affinity, low-capacity pathway of endocytosis. Megalin and cubilin work in concert to reabsorb a variety of filtered molecules (35, 37, 205, 208–210). Without this mechanism of retrieval and preservation, protein loss, malnutrition, vitamin deficiencies (B_12_ and folic acid), and other consequences would ensue. This again emphasizes the role of PTC transcytosis in preserving numerous ligands for continued availability to the organism.

Although both megalin (183) and cubilin (207) can bind albumin, megalin’s principal role in albumin reabsorption seems to be in catalyzing the retrieval and internalization of apical cubilin-albumin complexes from glomerular filtrate. Megalin binds to >60 different ligands, whereas cubilin binds to far fewer (71).

The capacity of the multireceptor retrieval system in mice is ∼30–50 g of albumin daily (211). Proteinuria and albuminuria occur when this system is reduced. Megalin knockout models have a rate of internalization of endogenous cubilin-bound albumin complexes that is markedly reduced. Urinary albumin excretion is increased approximately sixfold over wild-type mice in Cubilin-deficient mice (211). Humans also have increased albuminuria with cubilin mutations (212), though rarely does this reach nephrotic range, suggesting that additional mechanism(s) for albumin reabsorption exist. A genomewide association study in the general population found that microalbuminuric patients had a CUBN missense mutation (213). In two siblings with proteinuria reaching 2 g daily, exome sequencing identified a homozygous frameshift cubilin mutation associated with decreased PT albumin uptake (214). Interestingly, mice deficient in megalin and cubilin had similar amounts of albuminuria compared with mice with cubilin deficiency alone (211), suggesting that the principal role of megalin is to facilitate cubilin-albumin reabsorption in PTs.

**FIGURE 10.**
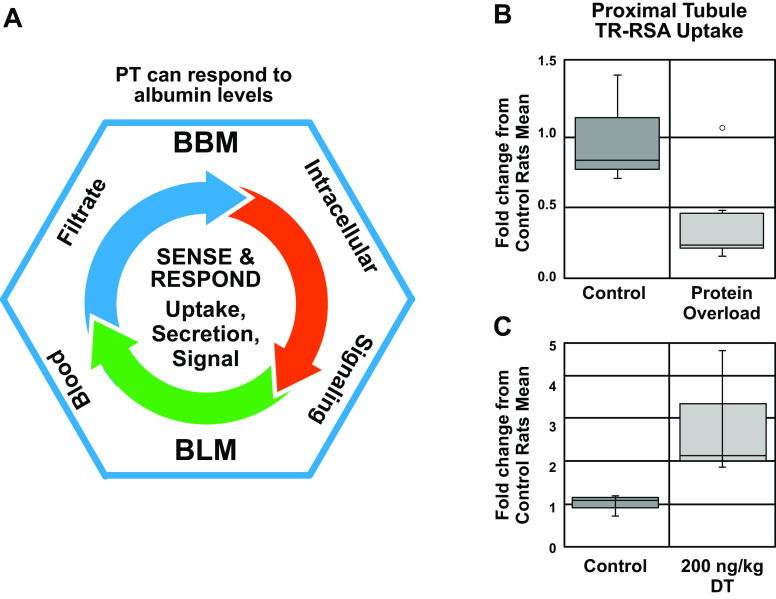
*A*: proximal tubules (PTs) determine the physiological state of the body by “sensing” urine and serum albumin levels. Proximal tubule cells (PTCs) can adjust uptake and secretion mechanisms to impact the physiological state both directly and indirectly. *B*: box plot showing quantification of Texas Red-X-rat serum albumin (TR-RSA) reabsorption by all surface PTs of control (*n* = 3 rats, 157 fields quantified) and albumin-overloaded (*n* = 8 rats, 176 fields quantified) rats. A significant reduction in albumin uptake (*P* < 0.01, KaleidaGraph, Student’s *t* test) was seen with albumin overloading. *C*: box plot showing the quantification of TR-RSA uptake in all surface PTs of control rats (*n* = 3 rats, 101 fields quantified) and rats treated with diphtheria toxin (DT) to increase glomerular albumin permeability (*n* = 3 rats, 106 fields quantified). There was a significant increase in PTC albumin uptake (*P* = 0.05, Student’s *t* test 1-tailed equal variance) when filtrate albumin concentration increased. Modified from Ref. 3, with permission from the *Journal of the American Society of Nephrology*.

Regulation of PT albumin uptake involves multiple proteins and is discussed below. We do know that PTs are able to respond to serum albumin levels (FIGURE 10*A*). Using the albumin overload model, Wagner et al. (3) showed that elevating serum albumin to abnormal levels caused the PTs to reduce their reabsorption of filtered albumin, resulting in increased albuminuria until serum levels had returned to normal (FIGURE 10*B*).

In these studies, neither glomerular filtration rate (GFR) nor GSCa changed. They also showed that if GSCa was increased by systemically injuring the podocyte with a podocyte-specific diphtheria receptor transgenic toxin model, both S1 and S2 PTCs dramatically increased albumin reabsorption. In this later model serum albumin was reduced to approximately half normal levels (FIGURE 10*C*). The ability for PT to increase albumin uptake when the glomerular permeability is increased was also shown by Ren et al. and resulted in increased lysosomal enzyme synthesis (215, 216).

## 8. DYSFUNCTIONAL PROXIMAL TUBULE CELLS AND SPECIFIC GENE MUTATIONS OR MANIPULATIONS CAN ALL LEAD TO ALBUMINURIA

Albuminuria has long been used as a marker of chronic kidney disease, whether originating from glomerular dysfunction or defective proximal tubule reabsorption. In many cases albuminuria is likely a combination of both glomerular and PT dysfunction (2, 3). A high filtered load of albumin resulting in increased reabsorbed albumin may well overload the capacity of the PT to effectively and safely handle the albumin load and lead to PT injury. The cellular mechanism(s) responsible for reduced or increased albumin uptake remains to be fully determined. However, disruption of numerous specific proximal tubule processes can cause proteinuria and albuminuria. TABLE 3 lists multiple conditions or gene alterations that result in albumin processing dysfunction and/or albuminuria.

**Table 3.** Key studies reporting albuminuria or albumin processing dysfunction

Gene, Protein, or Defective Agent/Treatment	Reference(s)
Endogenous or exogenous protein overload	(3)
Megalin-cubilin	(165, 205, 207, 213, 214, 218–222, 224)
Bardoxolone	(217)
NHE-3 (SLC9A3)	(225)
CLC-5	(226, 227, 229)
Rab 38	(230–232)
Statins	(233–235)
Diphtheria toxin-induced PTC injury	(236–238)
d-Serine induced PTC injury	(239)
GWAS: cubilin but also OAF and PRKCI	(251)

GWAS, genomewide association study; PTC proximal tubule cell.

These defects range from the multiligand endocytic receptor complex, megalin and cubilin, to drugs that alter expression of megalin, all suggesting the central role of cubilin and megalin in albumin reabsorption and catabolism (205, 207). The anti-inflammatory agent bardoxolone methyl causes significant albuminuria by decreasing PT megalin expression (217). Total body irradiation in rats reduces albumin and megalin binding to cubilin, causing albuminuria (218). Megalin-knockout mice were observed to have normal PT brush border membranes, but there was a reduction in number of coated pits, endosomes, and lysosomes (219). In these KO mice little difference in the amount of urinary albumin was found, though low-molecular-weight proteinuria occurred. The use of the Cre-LoxP system to individually knock out cubilin, megalin, or both revealed elevated albuminuria in both cubilin and megalin knockouts, although no additive effect was observed in the double knockouts, consistent with megalin not serving as a direct binding partner for albumin (211). Albuminuria in these models increased approximately sixfold. Mutations in megalin have been shown to cause Donnai–Barrow and facio-oculo-acoustico-renal syndromes, with all affected children exhibiting some level of proteinuria (220, 221). Analysis of cubilin mutations identified it as a gene locus for albuminuria (213, 214, 222) and a risk factor for end-stage renal disease in native kidneys and graft failure in transplant kidneys (223). In contrast, COOH-terminal variants are associated with chronic proteinuria and normal renal function (165). Cubilin heterozygous mice, with reduced expression of cubilin, have normal megalin levels but reduced albumin uptake by proximal tubule cells, significantly increased albuminuria, and a 17% decrease in plasma albumin (224). Exploration of these specific mutations will lead to a more complete understanding of the role for both of these receptors in the proximal tubule and in regard to albumin homeostasis.

A specific protein’s function is often revealed in part by creation of knockout mice and evaluation of specific mutations either found in nature or created. Knockout of the apical Na^+^/H^+^ exchanger isoform 3 (NHE3) resulted in albuminuria (225). Chloride channel 5 (CLC-5) mutations in apical endosomes in mouse models, as is also seen in Dent’s disease, demonstrate defective clathrin-mediated endocytosis and FPE, deficient endosomal acidification, internalization of the Na^+^-phosphate cotransporter 2 (NaPi-2) and NHE3, and increased proteinuria (226–228). Defective PT endocytosis in CLC-5-knockout mice is secondary to trafficking defects resulting in loss of brush border cubilin and megalin and causing albuminuria (229). Rab 38 dysfunction, or lack of function, in rats results in albuminuria by reducing albumin endocytosis without increasing glomerular permeability (230). The extent of proteinuria correlated with the Rab 38 mutation and not the fawn hooded hypertensive congenic rat mutation associated with increased albumin permeability (231, 232). Statins, by inhibiting guanosine triphosphatase prenylation, reduced proximal tubule endocytosis and enhanced proteinuria and albuminuria (233–235).

Rats with selective PT injury have marked and sustained dose-dependent albuminuria without associated glomerular injury or morphological alterations, requiring up to 35 days to recover after injection. This occurred in association with PT recovery. Studies by three independent investigators selectively poisoning PT or causing global PT dysfunction resulted in large quantities of albumin ending up in the urine. Transgenic mice selectively expressing the diphtheria receptor on PTs administered diphtheria toxin sustained a dose-dependent selective PT injury resulting in nephrotic-range proteinuria (236–238). Histological evaluation revealed no glomerular morphological changes that would account for this level of proteinuria and albuminuria (237). d-Serine, a selective PT toxin, showed similar results many years earlier (239). With recovery of PT function, proteinuria returned to baseline levels (237).

The role of the PT in progressive albuminuria in chronic kidney disease has not been thoroughly evaluated. Multiple lines of evidence suggest a role for reduced PT albumin reabsorption, although this has in large part been ignored (1). Many forms of nephrotoxic nephropathy result in albuminuria without glomerular alterations. In fact, albuminuria was one of the best urinary biomarkers of AKI in a large multi-nephrotoxin study in rats (240). Reductions in PTC albumin receptor expression have been reported in animal models and human chronic kidney diseases (241–243) including diabetes (244, 245) and hypertension (246). Increased urinary excretion of megalin and cubilin has been identified in models of Alport syndrome (247), in human diabetes mellitus (248, 249), and in IgA nephritis (250). In a dog model of progressive Alport syndrome there was increased urinary excretion of megalin and cubilin associated with their reduced PT content. Serial kidney biopsies revealed a duration-dependent reduction in PT content of low-, intermediate-, and high-molecular-weight proteins implying reduced uptake. This was associated with a progressively increasing urinary excretion of these proteins including albumin (247). Taken together, these observations imply that CKD-induced reductions in PT albumin receptors mediate reduced albumin reabsorption and increased excretion during CKD progression. This may well explain continued high and even increasing levels of albuminuria while GFR is declining. Additional potential endocytic gene loci affecting PT endocytosis and resulting in CKD were identified by a recent genomewide association study (251). Besides the well-known association between CUBN and microalbuminuria and macroalbuminuria, Out At First (OAF) and Protein kinase C iota (PRKCI) were identified as PT endocytosis markers. This was validated in knockdown of OAF and PRKCI orthologs in *Drosophila* nephrocytes resulting in a reduction of albumin endocytosis. Silencing fly PRKCI further impaired slit diaphragm formation, which would lead to increased albumin filtration.

## 9. TRANSCYTOSIS BY PROXIMAL TUBULE CELL

Endocytic cargo that undergoes transcytosis has been studied in multiple systems, and some common genes/proteins have been identified (87). These include Rabs, Snares, and both actin and microtubule components. Since albumin has been shown to be most avidly taken up in S1 PT, we focus on the transcytotic components concentrated in this segment. For instance, the SNARE VAMP-8 was necessary for polarized sorting and transcytosis. Transcytosis from the Rab11 CRE compartment involves Rab11-FIP5 (82, 87). There is also evidence that *N*- and *O*-glycans can act as sorting signals in transcytosis involving galectins (family of proteins with different glycan specificity) (87, 103). The role of glycosylation in regulating ligand-receptor interactions and regulating protein-protein interactions is expanding rapidly, and this posttranslational modification along with others, i.e., phosphorylation, need to be further investigated to better understand their respective impact (252, 253).

The only known transcytotic receptor for albumin is the neonatal Fc receptor (FcRn), which is a nonclassical member of the Fc gamma (γ) receptors whose structure is related to the major histocompatibility class I (MHC I) family (254). Both IgG and serum albumin have a marked prolonged serum half-life due to their interaction with FcRn that directs them away from the lysosomal degradation pathway and recycles them to the vasculature (255, 256). Albumin accumulation within early endosomes was shown to be independent of the affinity for FcRn, but differences in albumin binding affinities for FcRn altered the distribution of albumin between late endosomes and lysosomes. As the FcRn affinity of modified albumins increased, there was less lysosomal trafficking and increased recycling (257). Recombinant proteins with a FcRn binding site have been shown to undergo FcRn-mediated transcytosis and recycling (258).

FcRn is a heterodimeric integral membrane protein composed of an a-chain of the nonclassical MHC class I family and β2-microglobulin (259). It contains distinct and noninteractive binding sites for IgG and albumin, and its affinity for each is increased >100-fold in an acidic environment (260). Fatty acids bound to albumin are known to compete for the FcRn binding site, but their dissociation in the acidic pH of the endosome opens the site for FcRn binding (261).The crystal structure for albumin binding to FcRn has been determined, and increases in binding affinity increase the serum half-life of albumin (262, 263). The FcRn-IgG interaction has been studied extensively in multiple tissues, and studies support a pH-dependent increased ligand binding in endosomes that prevents ligand targeting to lysosomes, followed by ligand transcytosis and subsequent extracellular release (264–267). Studies have documented the presence of FcRn in the brush border (BB) of proximal tubule cells (3, 268), direct visualization of albumin transcytosis in proximal tubule cells intravitally (4), and molecular evidence for an active role of FcRn in proximal tubule albumin transcytosis (5, 269). Most FcRn has been shown to be in subapical endosomes, with only small amounts localized to the surface membrane in HepG2 cells (270). Given the pH of the glomerular filtrate, little binding of albumin to FcRn would be expected at the surface.

FcRn is derived from the FCGRT gene encoded on chromosome 19 and on chromosome 6 outside of the MHC class I locus. However, rat and mouse FcRn are encoded on chromosome 7 and are 91% identical. Human FcRn has one *N*-glycan moiety and has a molecular mass of 42–44 kDa, and rat FcRn’s molecular mass is 48–52 kDa. This difference is attributable to one and three additional *N*-glycan moieties, respectively (271). FcRn is located on numerous cell types throughout the body including endothelial cells; epithelial cells, hepatocytes, spleen, and lung; placental syncytiotrophoblasts; and monocytes, polymorphonuclear neutrophils, and dendritic cells (272–278). FcRn is found in several kidney cell types including podocytes, endothelial cells, proximal tubule cells, and cortical collecting duct cells (268). Immunofluorescence labeling studies of human kidney sections showed FcRn within PTC apical membrane and in endosomes (268).

FcRn has been shown in many cell types to transport albumin across surface membranes. This extends albumin’s life span for continued function (269). FcRn also plays a major role in initiating fetal immunity by transporting maternal IgG across the neonatal placenta and by transcytosis of IgG across neonatal intestinal cells (272, 279–281). Overexpression of FcRn in transgenic mice and rabbits has humoral immunity, resulting in a 3- to 10-fold increase in serum IgM and IgG. Interestingly, this also results in increased albumin concentrations (282, 283). Elevated immunoglobulin levels do not interfere with albumin processing, as the immunoglobulin and albumin binding sites are distinct and independent binding sites. FcRn mediates transcytosis and reclamation of IgG by PTs (284).

FcRn-mediated transcytosis has been studied and delineated in the small intestine. The mechanism begins with endocytosis via clathrin-coated pits, at low luminal pH (265, 273, 285–288). The equilibrium dissociation constant for albumin FcRn binding, *K*_D_, is markedly reduced at pH 7 (34–408 µM) and then at pH 5.0 (0.2–0.7 µM). Consequently, when albumin is internalized bound to the Megalin/Cubilin complex, or in fluid-phase endosomes, it traffics to the late endosomes and encounters acidic pH. At this point a “handoff” of albumin to the FcRn receptor occurs, thus directing it down the transcytotic pathway. When the transcytotic vesicle fuses with the plasma membrane, it encounters neutral physiological pH mediating a rapid dissociation of albumin from FcRn. After release into the interstitial fluid space, FcRn mediates transcytosis across endothelium. PT FcRn receptor is recycled back to the apical membrane or apical endosomal compartment ready for another cycle of albumin and or IgG transcytosis. Data for IgG transport exist in several other cell types including pneumocytes (286), skeletal muscle endothelium, skin endothelium (273, 287), central nervous system endothelium, and the choroid plexus (288).

The functional significance of PT FcRn was shown in several transgenic studies. FcRn-knockout mice have a marked reduction in the half-life of plasma albumin, and plasma concentrations are reduced by ∼50%, resulting from greater catabolism and albumin clearance (255, 269, 289). FcRn-knockout mice had increased quantities of albumin at the PT apical membranes and increased fractional excretion of albumin (269). Kidneys from FcRn-knockout mice, transplanted into wild-type mice, resulted in a decline of serum albumin to 40–50% of baseline over a 3-wk period. Conversely, serum albumin concentrations increased when wild-type kidneys were transplanted into FcRn-knockout mice (269). Additionally, mice with FcRn or β2-microglobulin mutations had a reduction in serum half-lives for both IgG and albumin (289).

**FIGURE 11.**
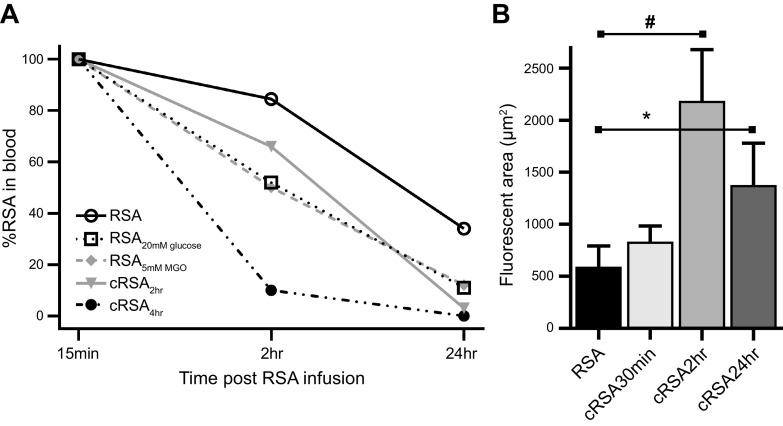
*A*: vascular clearance of wild-type and modified rat serum albumins (RSAs) (6, 155). Fluorescently tagged (Texas Red-X or Oregon Green-X) RSA and one of the modified RSAs were injected simultaneously into the same rat, and blood was collected after injection at 15 min, 2 h, and 24 h. Each albumin was evaluated in 4 rats (male Sprague-Dawley rats, 180–220 g) that received both a control and a modified albumin. The 15 min collection time point was set to 100%, and the decrease in fluorescence followed at 2 h and 24 h. Note that modified albumins all had increased vascular clearance. GraphPad Prism was used to graph means ± SD for each 2 and 24 h time point (6, 155). cRSA, carbamylated RSA; MGO, methylglyoxal. *B*: albumin in kidney endothelial/interstitial regions (6). Quantification of albumins in kidney endothelial/interstitial regions showed significant increases in accumulation between RSA and albumin modified with potassium cyanate for 30 min, 2 h, or 4 h (cRSA_2hr_ #P < 0.05 and RSA vs. cRSA_4hr_ *P < 0.05) (6). Images from Refs. 6 and 155, with permission from the American Physiological Society.

**FIGURE 12.**
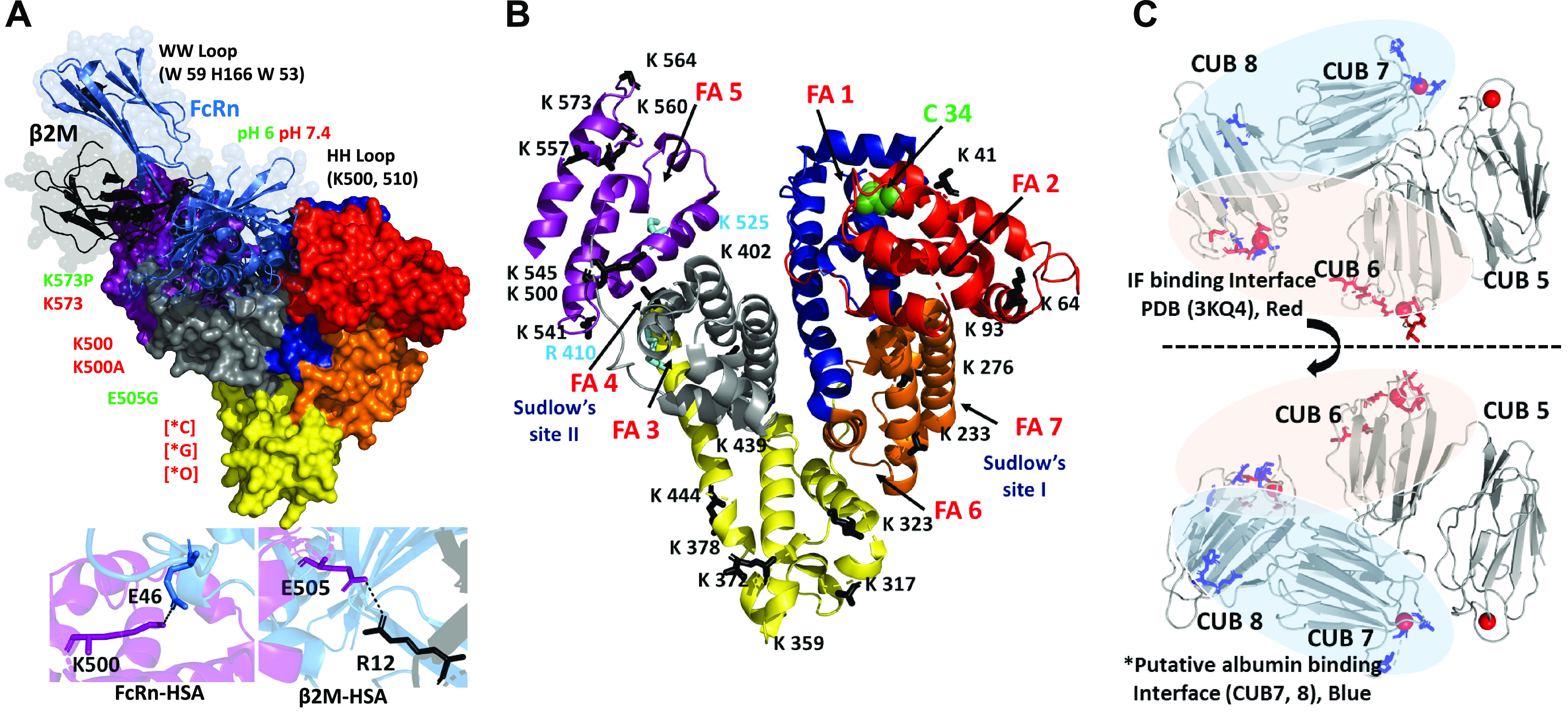
*A*: 3-dimensional structural view of human serum albumin (HSA)-neonatal Fc receptor (FcRn) complex (PDB ID 4N0F). HSA (DI, DII, and DIII), FcRn α-chain and β2-microglobulin (β2M) are shown in light blue and black, respectively (296). Albumin domains are colored as in FIGURE 5: red, IA; blue, IB; light brown, IIA; yellow, IIB; gray, IIIA; purple, IIIB. Green color highlights key amino acid changes and conditions that favor FcRn-albumin interaction (297, 298). Red color highlights amino acid mutations and conditions that destabilize FcRn-albumin interaction including carbamylation (*C), glycation (*G), oxidation (*O), and pH (116, 299, 300). The *inset* highlights 2 salt bridges critical for FcRn-HSA and β2M-HSA interactions that are affected by modifications (296). *B*: albumin structure (PDB ID 1E78) labeled for many of its various binding moieties and modifications including carbamylation, glycation, fatty acid (FA), oxidation, and Sudlow’s I and II sites known for binding different drugs, metabolites, and metal ions. The primary site of both glycation and carbamylation is K525 (blue), whereas R410 is a non-lysine glycated site and C34 is the oxidation site (6, 116, 155, 299–303). Other sites are noted to emphasize the potential impact of modifications/associations on albumin’s many interactions. *C*: structure of the CUB5–8–IF–Cbl complex (PDB ID 3KQ4) (301) is presented in 2 different views. The concave interface (red) is required for the intrinsic factor (IF) interaction, which takes place mainly via Cub6 and Cub8 domains. The convex surface of Cub5–8 (blue) binds to albumin via interactions with mainly Cub7 and Cub8 residues. This is based on our cross linking mass spectrometry and docking studies (unpublished observations). Note that the albumin binding interface is distinct from IF binding site. The red balls designate Ca^2+^ binding sites.

Over 20 years ago the Ward laboratory established that there were critical differences across species of FcRn-antibody affinity (290). Most notable, human FcRn bound very weakly to mouse IgG. Species differences in FcRn-albumin binding were later reported (155, 291). The development of therapeutic antibodies has utilized this information to design novel immunoglobulins with modified FcRn binding properties to improve effectiveness (292, 293). The recognition that albumin-FcRn affinity can also be impacted by species differences and thus utilized to extend or reduce half-life of bound albumins has more recently been investigated (155, 254, 294). Of critical importance for understanding albumin dynamics may be how modified albumins, i.e., glycated, carbamylated, oxidized, and albumin bound to molecules such as drugs or fluorophores, affect the albumin-FcRn pH-dependent binding interaction (TABLE 4). Recent studies have documented that glycated and carbamylated albumin have reduced binding to FcRn (6, 155). In addition, vascular clearance is increased as binding to FcRn is reduced (FIGURE 11*A*), and evidence for interstitial/endothelial kidney accumulation of the modified albumins was observed (FIGURE 11*B*). Not surprising yet underappreciated was the documentation of reduced binding to FcRn (TABLE 5) and increased vascular clearance of albumin when fluorophores were conjugated at ratios much greater than 1:1 (155). This emphasizes the importance of understanding the effects of labeling on a protein’s subsequent protein interactions. Note that the Howard laboratory has also designed a recombinant human albumin with multiple surface thiols for stable conjugation that retain FcRn binding (295). We have also observed that albumins with decreased binding to FcRn can have reduced cubilin binding (unpublished observation). FIGURE 12 presents the structural interaction sites of FcRn-human serum albumin (HSA) (FIGURE 12*A*), CUB5-8-HSA along with our unpublished CUB7,8-RSA sites (FIGURE 12*C*), and an HSA diagram with most of these interaction sites noted (FIGURE 12*B*).

**Table 4.** Assessment of rFcRn binding to albumins and lgGs

Ligand	rFcRn pH 6 *K*_d_, µM	hFcRn pH 6 *K*_d_, µM	mFcRn pH 6 *K*_d_, µM
*Albumins*			
Rat	11.6	∼1.1	SB
Rabbit	11.8		
Human	>100	4.6–5.2	∼86
Human	WB	1.1	WB
Porcine	>100	>50	
Mouse	>100	0.8	9.3–SB
Bovine	>100		
Sheeo	>100		
Rhesus	WB		
*IgGs*			
Rat	1.8		∼0.45
Rat lqG2a	0.014	NB	
Rabbit	0.19	SB	SB
Human	0.63	0.70	0.265
	0.03	0.09	
Human	0.05	2.35	1.2
Human laG1		0.63	0.082
Bovine	1.5	WB	SB
Mouse	>100	WB	∼0.01
Mouse lqG1		>50	0.75
Mouse lqG2a	0.019	NB	0.025
Rat and Mouse			
lqGs	∼0.05		

FcRn, neonatal Fc receptor; hFcRn, human FcRn; *K*_d_, dissociation coefficient; mFcRn, mouse FcRn; NB, no binding; rFcRn, rat FcRn; SB, strong binding; WB, weak binding.

**Table 5.** Assessment of fluorescent dye-albumin conjugates on FcRn binding

Fluorophore Conjugate (ratio of dye to albumin)	pH 6 *K*_d_, µM	pH 7.4 *K*_d_, µM
CF-594 maleimide (1:3)	1.5	NB
Texas Red-X (1:1)	5.4	NB
Texas Red-X (4:1)	>40	NB
Alexa 488 (4.5:1)	NB	NB
Fluorescein (6.4:1)	69	1.0
Alexa 568 (4:1)	17	NB
Texas Red-X (2:1)	10	NB
Texas Red-X (5:1)	>100	NB
Texas Red (1:1)	6	11
Alexa 647 maleimide (1:1)	11	NB
DO BSA	NB	NB
No dye	11.6	>100

*K*_d_, dissociation coefficient; NB, no binding; DQ BSA, dye quenched bovine serum albumin.

Given these interactions, modifications to albumin that reduce binding to FcRn may mediate lysosomal trafficking while “normal” albumin undergoes transcytosis (2). This would in effect be a physiological intracellular molecular sorting mechanism to reduce circulating and potentially toxic albumin molecules while retaining normal albumin. This may be particularly important in patients with diabetes or CKD, where increased glycation and carbamylation of albumin, associated with high glucose or urea levels, could lead to overloading of lysosomes and a reduced serum half-life of albumin. This could result in specific endothelial or PT inflammation and injury. In this regard, both glycated and carbamylated albumin are known to be risk factors for mortality (115, 299, 304, 305). This hypothesis is supported by reduced cellular release of internalized albumin and the rapid turnover of modified albumins (283).

### 9.1. Functional Significance of PT Reclamation via FcRn

Many studies have addressed the mechanism of FcRn trafficking, often in the context of IgG transcytosis (306). Whether similar mechanisms occur in vivo for the handling of albumin remains to be determined. Studies in MDCK cells stably expressing FcRn receptor showed that transcytosis of IgG moved through EEA1- and Rab11-positive vesicles and the CRE believed to be the site of Megalin-to-FcRn handoff (307). Delivery of FcRn and its cargo to apical or basolateral surface was also found to be dependent upon microtubules. Studies in human dermal microvascular endothelial cells (HMEC-1) showed a dynamic role for Rabs 4 and 11, Appl1, and SNX4 in tubulovesicular transport carriers coming to and from common sorting endosomes (308) in addition to showing that albumin with reduced FcRn binding had increased localization to the lysosome (257). Finally, a targeted RNA interference (RNAi) screen identified 23 genes whose suppression decreased FcRn-mediated transcytosis. Understanding the impact of these disruptions in vivo will be important (309). Other in vitro cell studies have shown that *1*) FcRn exits sorting endosomes in Rab4 and Rab11 compartments, with Rab11 being present at exocytosis (89); *2*) the FcRn cytoplasmic tail contains serine phosphorylation sites and an adaptor protein-2 binding site, both necessary for apical-to-basolateral trafficking (310–312); and, in addition, 3) calmodulin and FcRn glycosylation may regulate FcRn directional transport (313, 314).

In conclusion, multiple mechanisms of membrane, receptor, and cargo have been defined for cells, and although CME is very important, and the most studied, other mechanisms are increasingly being shown to have an important role. Further studies, investigating the highly polarized and specialized epithelial cells such as proximal tubules in vivo are necessary to fully understand the specific pathways and compartments traversed by cargo when internalized.

## 10. ALBUMIN AS A THERAPEUTIC HALF-LIFE-EXTENDING AGENT

Molecular selection technologies are being used to generate novel therapeutically promising peptides, scaffold proteins, and radioligands targeted toward specific receptors, signaling molecules, and tumor surface antigens. However, rapid elimination from the plasma, due to their small size and kidney filtration, and nephrotoxicity limit their potential as therapeutic agents. To combat these limitations, strategies have been developed to improve the pharmacokinetic characteristics of these potential therapeutics (263, 315). Both noncovalent and genetic fusion to albumin have been tried (316). As is shown in FIGURE 6, a number of drugs bind to albumin under physiological circumstances, and these binding domains can be exploited for use by other agents. Multiple fusion peptides have been developed. Successful fusion proteins must bind to albumin without inducing any conformational changes, remain bound to albumin in an acidic environment, and not induce reductions in albumin binding to FcRn. These characteristics ensure FcRn-mediated transcytosis of the albumin-bound therapeutic agent. If the agent bound to albumin dissociates in the acidic pH of the endosome, then the plasma half-life is not extended and toxicity may occur in PT. Another approach has been to minimize the albumin binding domain (ABD) necessary for FcRn binding in an attempt to increase cellular penetration while maintaining the long plasma half-life of the therapeutic agent (263, 317). A minimal albumin binding domain, derived from streptococcal protein G, resulted in efficient half-life extension of proteins by indirect targeting of FcRn resulting in transcytosis. As an example of the power of this approach, an albumin binding domain with high affinity for human serum albumin was used to circumvent the short half-life of Glucagon-like peptide-1 (GLP-1), a promising peptide for the treatment of type 2 diabetes mellitus. Three different ABD-fusion GLP-1 proteins bound HSA with high affinity, extended its half-life, and improved the glucose-lowering effect of GLP-1 (318). Studies like this have important implications for altering in vivo pharmacokinetics of peptides and proteins to improve pharmacodynamic and pharmacokinetic properties. Interestingly, low expression in tumor cells has been shown to limit recycling of albumin, leading to enhanced growth by increasing tumor cell consumption of albumin (319).

## 11. UTILITY OF RAP AS AN EXTRACELLULAR AGENT TO BLOCK RECEPTOR-LIGAND INTERACTION

RAP was first identified as a 39-kDa protein copurifying with the LRP receptor and shown to inhibit LRP ligand binding (320). It was shown to be a critical chaperone in the biosynthetic pathway of LDL receptor family members including megalin (186). Multiple studies have utilized RAP to inhibit ligand binding to megalin or other LDL receptors both in vitro and in vivo. Ligands that have had their uptake inhibited by RAP in cultured cells include myeloma light chains (321) and transferrin (322). In vivo studies have shown inhibition of albumin (183), reduced Cd-metallothionein toxicity (323), altered subcellular distribution of the type II sodium-phosphate cotransporter (324), and accumulation of zymogen form of matrix metalloproteinases and tissue inhibitor of metalloproteinases (325). Evidence suggests that RAP may cause an acute loss of megalin from the BBM (323), and although RAP inhibits multiple ligand-LDL receptor interactions, whether it impacts indirectly or directly other membrane proteins or trafficking pathways has not been explored.

## 12. CONCLUSIONS, FINAL REMARKS, AND FUTURE PERSPECTIVES

The role of the PT in albumin reabsorption and transcytosis is an established area but often downplayed as a source of albuminuria and cellular inflammation. Numerous studies support this role including single PT genetic alterations in rodents and human diseases, knockout mouse models, two-photon microscopy, and tissue clearing studies. Complete PT dysfunction results in a high level of albuminuria without increased glomerular contributions to the filtered load. The PT can also regulate albumin reabsorption, increasing or decreasing as needed, although the mechanism of this regulation needs further investigation. This regulation could be at the level of receptor surface expression, receptor posttranslational modification, or alteration to one of the many intracellular endocytic accessory proteins. Reabsorption of filtered albumin involves a high-affinity, low-capacity cubilin-megalin receptor-mediated process and a low-affinity, high-capacity fluid-phase endocytosis process. Deciphering how modified or altered albumins utilize these or other endocytic processes requires further investigation. Data on the transcytotic receptor FcRn within the PT cell suggest that its principal function is in pH-mediated binding, sorting, and intracellular trafficking between transcytosis and degradation pathways similar to IgG in other cells. Albumin that binds to FcRn is transcytosed, whereas “altered” albumin that does not bind goes to lysosomes for degradation. A question that needs investigation is whether, under certain physiological conditions, an increase in catabolism of normal albumin is required and, if so, whether this is regulated at the level of FcRn binding. Pathological processes including glycation, carbamylation, oxidation, and the binding of certain drugs to albumin reduce FcRn binding affinity, resulting in subsequent trafficking to the lysosome. Such a mechanism of selective processing and sorting is critical in reclaiming “normal” albumin via transcytosis and in the lysosomal catabolism of chemically altered and potentially toxic albumin forms. Whether altered albumin can trigger damage in the kidney or other tissues, and by what mechanism(s), will continue to be an important area of investigation. Binding of some therapeutic agents to albumin delivers them to the PT, where injury and AKI or CKD can result. What researchers must recognize is that albumin’s structure is altered by multiple modifications and ligand binding that can impact, sometimes in a species-dependent fashion, albumin’s ability to bind receptors responsible for its metabolism. Thus, molecular modeling and development of specific albumin mutants, albumin binding domains, or molecules to target specific albumin binding sites to minimize or maximize albumin binding, or associated molecule metabolism, are areas of active and important future investigation.

## SUPPLEMENTAL MATERIAL

10.6084/m9.figshare.14665968 Supplemental material can be found at https://doi.org/10.6084/m9.figshare.14665968.

## GRANTS

The authors acknowledge grant support to B.A.M. from the National Institutes of Health (DK 091623 and 079312) and support from the Department of Veterans Affairs through a Merit Review award.

## DISCLOSURES

No conflicts of interest, financial or otherwise, are declared by the authors.

## AUTHOR CONTRIBUTIONS

B.A.M. and M.C.W. drafted manuscript; B.A.M. and M.C.W. edited and revised manuscript; B.A.M., R.M.S., S.P.S.Y., and M.C.W. approved final version of manuscript.
